# Alcohol and substance-induced disorder treatment cases in mental health and addiction treatment facilities from 2013 to 2022: A descriptive study

**DOI:** 10.1371/journal.pmen.0000706

**Published:** 2026-09-02

**Authors:** Orrin D. Ware

**Affiliations:** School of Social Work, University of North Carolina at Chapel Hill, Chapel Hill, North Carolina, United States of America; PLOS: Public Library of Science, UNITED KINGDOM OF GREAT BRITAIN AND NORTHERN IRELAND

## Abstract

Substance use disorders may arise when individuals use substances to cope with adverse mental health symptoms. Conversely, mental health symptoms can develop because of substance use, a category of conditions called substance-induced disorders. This study used two United States-based datasets, the Mental Health Client-Level Data and the Treatment Episode Data Set Admissions, to summarize the number of cases with “alcohol-induced disorders” and “substance-induced disorders” from both mental health and substance use disorder treatment programs from 2013 to 2022. The primary study aim was to identify the percentage of cases with alcohol-induced disorders or substance-induced disorders in these treatment settings. Among all mental health disorder treatment cases, those with an alcohol or substance-induced disorder totaled N = 439,932, making up 0.7% of total cases over the analytic period. During the same period, alcohol or substance-induced disorders totaled N = 459,817, accounting for approximately 2.7% of all substance use disorder treatment cases. The majority (79%) of cases with an alcohol-induced disorder in substance use disorder treatment were identified as reporting alcohol use, with 68% having alcohol listed as their primary substance. Heroin and other opiates and synthetics accounted for the primary substance among 28.8% of cases with a substance-induced disorder in the substance use disorder treatment data. Approximately 1 in 3 cases with an alcohol-induced disorder and 1 in 4 cases with a substance-induced disorder had a depressive disorder diagnosis in mental health treatment. When considering the primary diagnosis, approximately 1 in 4 cases with an alcohol-induced disorder and 15% of cases with a substance-induced disorder had a depressive disorder as the primary diagnosis in mental health treatment. Substance-induced disorders are a significant public health concern, and more research is needed to better understand the clinical needs of this population.

## Introduction

Substance-induced disorders are a category of conditions caused by substance use [1]. Examples include intoxication, withdrawal, and mental health issues [1]. For instance, substance-induced depressive disorder occurs when substance use triggers depressive symptoms that meet the criteria for a depressive disorder [2–4]. Mental health symptoms resulting from substance-induced disorders are particularly unique, as clinicians must address both the emerging psychological issues and the underlying substance use. For example, in the management of substance-induced psychosis, it is recommended that the treatment address the symptoms of psychosis and the substance(s) of concern [5–12].

Facilities that treat mental health or substance use disorders may encounter patients with substance-induced disorders. This occurs because these conditions can show symptoms of both mental health and substance use disorders. However, the literature lacks descriptive studies on substance-induced disorder cases in both mental health and substance use disorder treatment settings. Data on substance-induced disorder comorbidities, such as mental health and substance use disorders, are also lacking. Descriptive studies are important to the research literature as they can highlight the prevalence of specific conditions and who is affected by them [13,14]. A study analyzing the Substance Abuse and Mental Health Services Administration’s Treatment Episode Data Set Admissions [15], reviewed all cases identified as having a “substance-induced disorder” from 1992 to 2022 [16]. However, the study excluded cases with an “alcohol-induced disorder” except where trends of these diagnoses were examined [16]. Given this, a study describing cases of “alcohol-induced disorder” in the substance use disorder treatment setting is needed. Additionally, no study has examined national data on alcohol or substance-induced disorders in the mental health treatment setting alongside substance use disorder cases in the United States. Building on Fedotov and Shustov’s previous work [16], this study described instances of “alcohol-induced disorders” and “substance-induced disorders” using national treatment data from both mental health and substance use disorder programs in the United States. This study also examines mental health diagnoses reported in those receiving mental health treatment. Further, it assesses substances used among individuals with an alcohol or substance-induced disorder between 2013 and 2022. Three aims guided this research study: [a] Identify the percentage of cases identified as having alcohol-induced disorders or substance-induced disorders in mental health and substance use disorder treatment settings, [b.] Examine the prevalence of other mental health diagnoses, such as anxiety and depressive disorders, among cases with alcohol-induced disorders or substance-induced disorders in mental health treatment, and [c.] Examine the prevalence of substances used, such as alcohol and cannabis, among cases with alcohol-induced disorders or substance-induced disorders in mental health treatment.

## Materials and methods

### Ethics statement

The University of North Carolina at Chapel Hill Institutional Review Board determined that this study does not meet the criteria for human subjects research. Further, participant consent is not applicable to this study.

### Data

This study utilized the Mental Health Client-Level Data [17] and the Treatment Episode Data Set Admissions [15]. Both are publicly available datasets provided by the Substance Abuse and Mental Health Services Administration. The Mental Health Client-Level Data includes records of individuals who received mental health treatment in the United States at facilities reporting their data to a state or other jurisdictional government [17]. The Treatment Episode Data Set Admissions contains records of treatment episodes for substance use disorder treatment admissions from facilities that receive public funding [15]. Both datasets are de-identified and offer annual cross-sectional data. The earliest year of available data for the Mental Health Client-Level Data is 2013; therefore, the analytic period for this study spans from 2013 to 2022.

### Sample selection

The Mental Health Client-Level Data and the Treatment Episode Data Set Admissions both include a variable that indicates whether a case has an “alcohol-induced disorder” or a “substance-induced disorder.” The variable in the Mental Health Client-Level Data is “SUB”. The variable in the Treatment Episode Data Set Admissions is “DSMCRIT.” These values are mutually exclusive, meaning a case can only have one of these two disorders listed in the dataset. All cases with either an “alcohol-induced disorder” or a “substance-induced disorder” from both datasets were selected and included in this current study. This resulted in N = 439,932 cases from the Mental Health Client-Level Data (2013–2022) and N = 459,817 cases from the Treatment Episode Data Set Admissions (2013–2022). Although substance-induced disorders can be diagnostically defined as conditions caused by the use of substances, including alcohol, since both datasets have values for “alcohol-induced disorder” and “substance-induced disorder,” both categories were separately examined in this study.

### Analysis

This descriptive study used univariable statistics to analyze demographic characteristics such as age, gender, and race/ethnicity among cases in both datasets. Annual counts of alcohol-induced disorder cases, substance-induced disorder cases, and total alcohol or substance-induced disorder cases across both datasets were examined. Since the Mental Health Client-Level Data focuses on mental health treatment, it includes a variable that identifies the primary mental health disorder. Similarly, the Treatment Episode Data Set Admissions, which centers on substance use disorder treatment, contains a variable that identifies the primary substance used. Accordingly, the percentages of primary mental health disorders were analyzed for mental health treatment cases, and the percentages of primary substances were examined for substance use disorder treatment cases. In addition to primary mental health disorders and primary substances, mental health disorders and substances used, as described in the respective datasets, were also examined. Up to three mental health disorders and substances used can be reported for each case in the Mental Health Client-Level Data and Treatment Episode Data Set Admissions, respectively. Therefore, while cases may have a listed primary mental health disorder or primary substance, secondary or tertiary substances may also be present. This study used IBM SPSS Version 31 [18] and R Version 4.5.1 [19], with ggplot2 to create figures [20].

## Results

### Mental health and substance use disorder treatment cases

**Fig 1:**
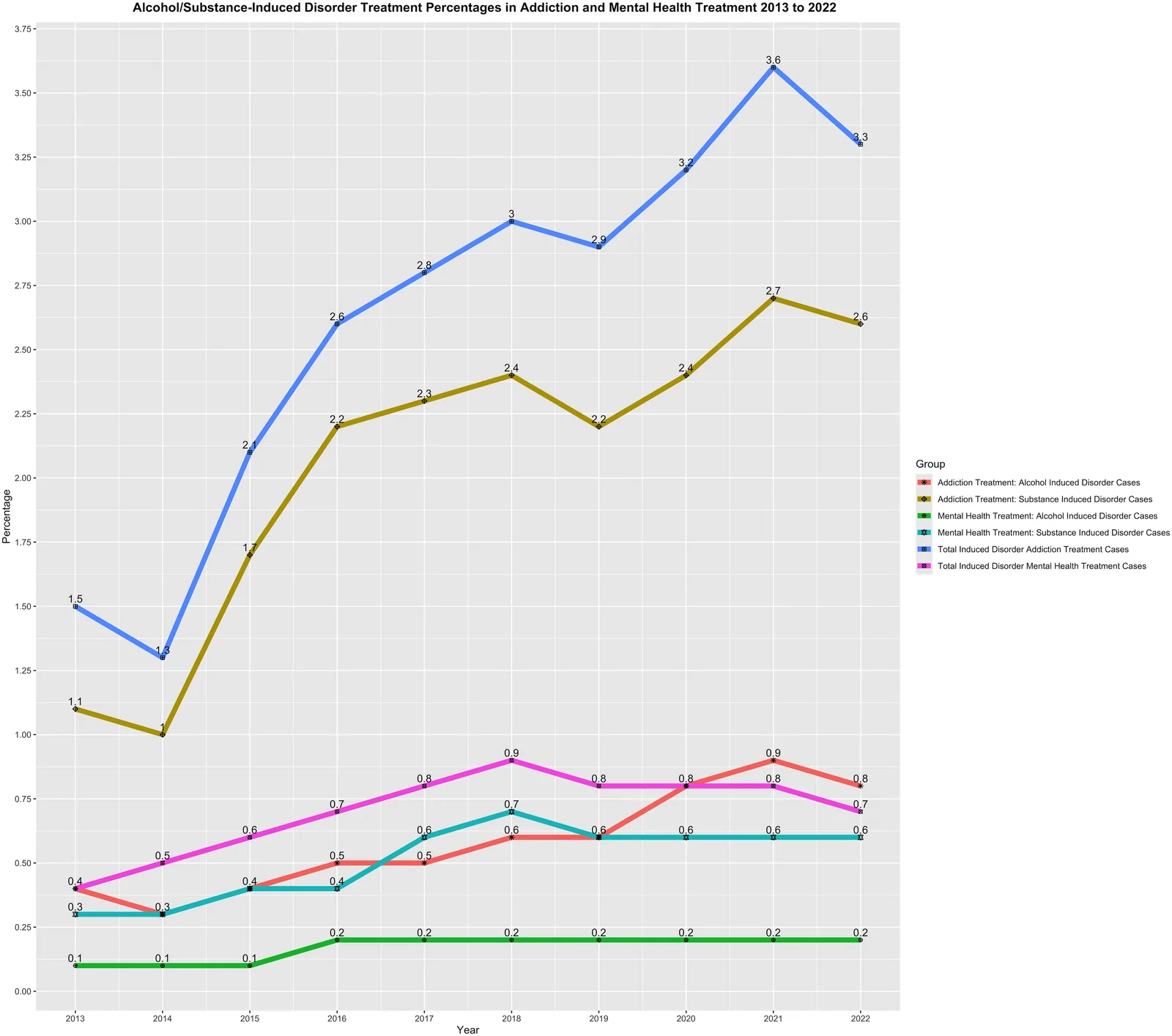
Alcohol/Substance-Induced Disorder Treatment Percentages in Addiction and Mental Health Treatment 2013 to 2022.

Combining the total 2013–2022 Mental Health Client-Level Data, including cases without an alcohol or substance-induced disorder, resulted in 61,589,330 cases. Alcohol-induced disorder cases accounted for 0.2% and substance-induced disorder cases for 0.5% of the total combined mental health treatment data. Similarly, combining the total 2013–2022 Treatment Episode Data Set Admissions data, including cases without an alcohol or substance-induced disorder, resulted in 17,445,707 cases. Alcohol-induced disorder cases accounted for 0.6% and substance-induced disorder cases for 2.1% of the total combined substance use disorder treatment data. Fig 1 provides the annual percentage of cases with an alcohol or substance-induced disorder across all cases in the mental health and substance use treatment datasets.

The mental health treatment data of selected alcohol or substance-induced disorder cases included N = 439,932 cases, with n = 111,720 (25.4%) alcohol-induced disorder cases and 328,212 (74.6%) substance-induced disorder cases. The addiction treatment admission data of selected alcohol or substance-induced disorder cases included N = 459,817 cases, with n = 100,331 (21.8%) alcohol-induced disorder cases and 359,486 (78.2%) substance-induced disorder cases. Demographic characteristics such as age, gender, and race and ethnicity may be found in Table 1.

**Table 1:** Characteristics of Addiction and Mental Health Treatment Cases with Substance-Induced Disorders.

Addiction Treatment Admissions; N = 459,817				
	Alcohol-Induced Disorders; n = 100,331		Substance-Induced Disorders; n = 359,486	
Demographics	n	%	n	%
**Age**				
12 to 17 Years Old	3,246	3.2%	13,043	3.6%
18 to 29 Years Old	23,601	23.5%	121,288	33.7%
30 to 39 Years Old	26,517	26.4%	112,565	31.3%
40 to 49 Years Old	21,155	21.1%	62,841	17.5%
50 Years and Older	25,812	25.7%	49,749	13.8%
**Gender**				
Female	32,412	32.3%	130,546	36.3%
Male	67,881	67.7%	228,667	63.6%
Missing	38	0.0%	273	0.1%
**Race and Ethnicity**				
Black or African American	21,907	21.8%	59,362	16.5%
Hispanic of Any Race	7,329	7.3%	42,961	12.0%
White	56,680	56.5%	206,265	57.4%
Another Race	6,132	6.1%	14,406	4.0%
Missing	8,283	8.3%	36,492	10.2%
**Mental Health Treatment Cases; N = 439,932**				
	**Alcohol-Induced Disorders; n = 111,720**		**Substance-Induced Disorders; n = 328,212**	
**Demographics**	**n**	**%**	**n**	**%**
**Age**				
0 to 17 Years Old	2,822	2.5%	18,490	5.6%
18 to 29 Years Old	18,876	16.9%	99,656	30.4%
30 to 39 Years Old	24,365	21.8%	95,267	29.1%
40 to 49 Years Old	24,389	21.9%	59,122	18.0%
50 Years and Older	41,152	36.9%	55,167	16.8%
**Gender**				
Female	40,284	36.1%	131,926	40.2%
Male	71,150	63.7%	195,327	59.5%
Missing	286	0.3%	959	0.3%
**Race and Ethnicity**				
Black or African American	15,668	14.0%	50,013	15.2%
Hispanic of Any Race	12,240	11.00%	39,641	12.10%
White	62,247	55.70%	178,598	54.40%
Another Race	6,474	5.80%	18,324	5.60%
Missing	15,091	13.50%	41,636	12.70%

Percentages are column percentages.

**Fig 2:**
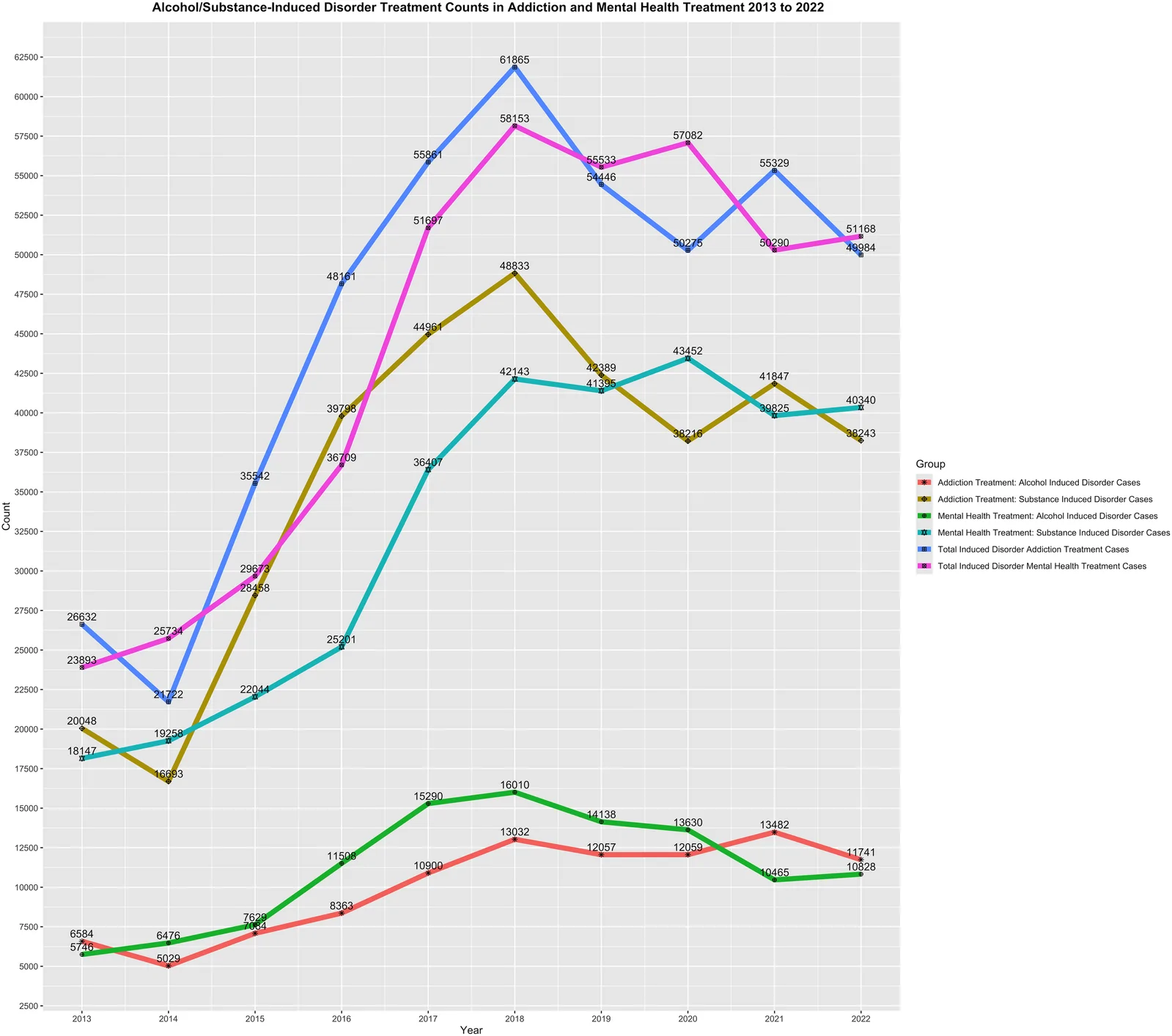
Alcohol/Substance-Induced Disorder Treatment Counts in Addiction and Mental Health Treatment 2013 to 2022.

Annual count data among all cases with alcohol or substance-induced disorders may be found in Fig 2. Examining Fig 2 shows that the total number of annual alcohol or substance-induced disorder diagnoses was higher in addiction treatment cases when observing the counts of mental health treatment cases, apart from the years 2014, 2019, 2020, and 2022. Also seen in Fig 2 is that cases with an alcohol-induced disorder had a lower count annually when observing the counts of substance-induced disorder.

### Substance use disorder treatment cases: Substances used

**Fig 3:**
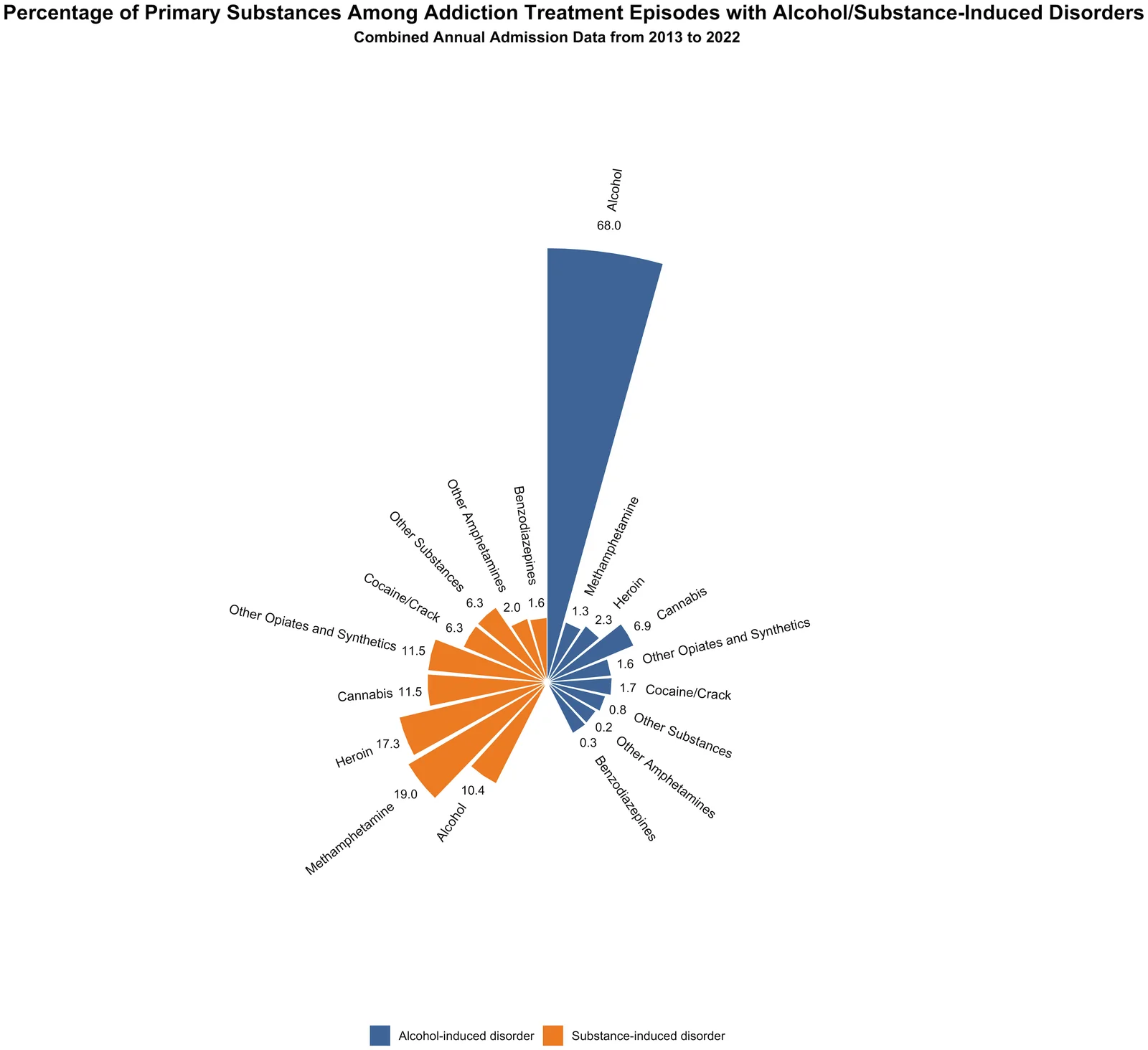
Percentage of Primary Substances Among Addiction Treatment Episodes with Alcohol/Substance-Induced Disorders. Combined Annual Admission Data from 2013 to 2022”.

The percentage of primary substances among cases with both alcohol and substance-induced disorders receiving treatment in the substance use disorder setting may be found in Fig 3. Alcohol was identified as the primary substance in 68.0% (68,186 / 100,331) of cases with an alcohol-induced disorder in substance use disorder treatment. Among cases with a substance-induced disorder in substance use disorder treatment, 19.0% (68,465 / 359,486) had methamphetamine as a primary substance, and 17.3% (62,117 / 359,486) had heroin as a primary substance. However, when heroin and other opiates and synthetics were combined, they accounted for the primary substance among 28.8% of cases with a substance-induced disorder.

**Fig 4:**
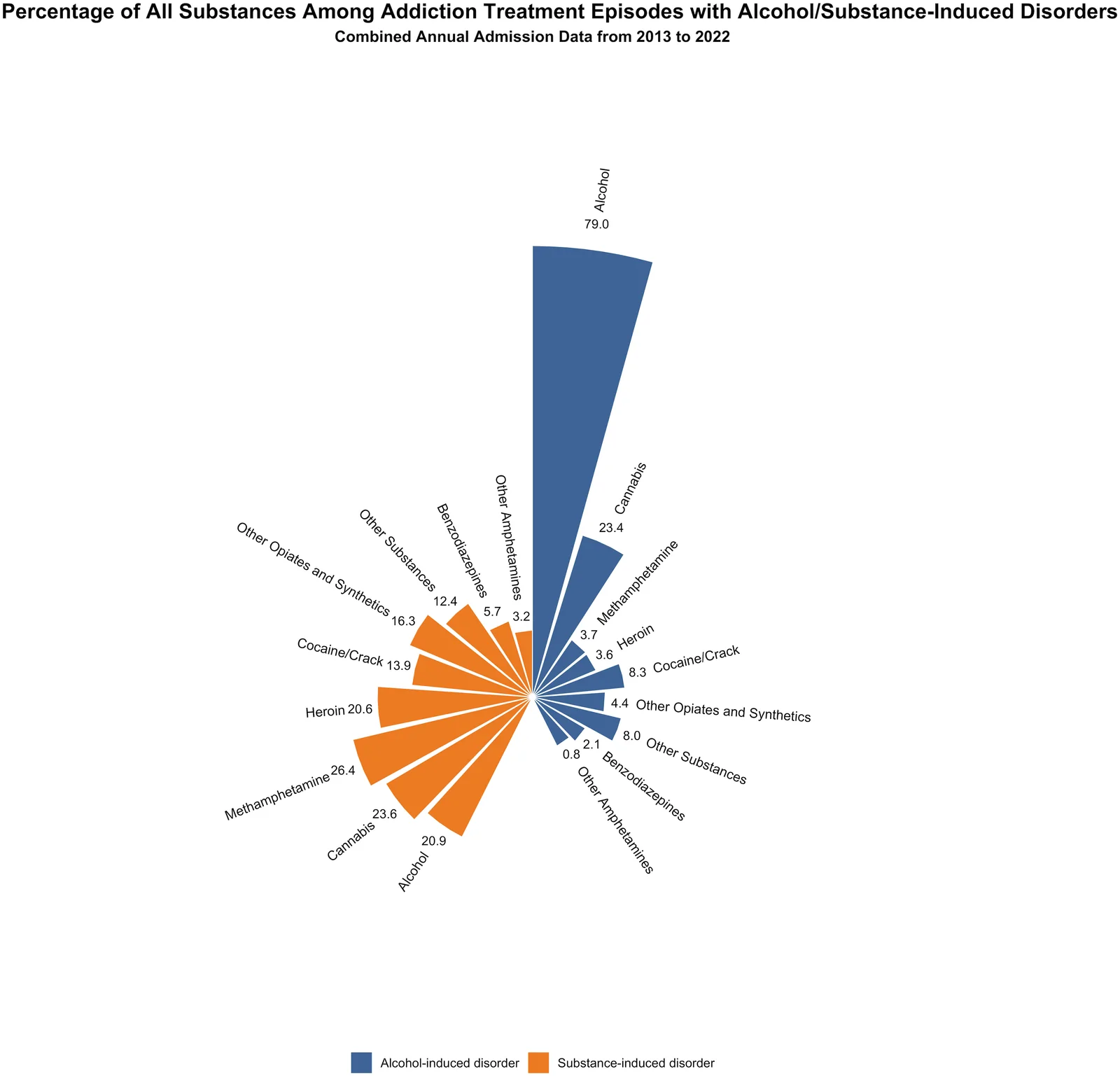
Percentage of All Substances Among Addiction Treatment Episodes with Alcohol/Substance-Induced Disorders. Combined Annual Admission Data from 2013 to 2022”.

As some cases also had a secondary and/or tertiary substance listed, not just a primary substance, Fig 4 shows cases with any substance reported in the substance use disorder treatment setting. Alcohol was identified as being used in 79.0% (79,290 / 100,331) of cases with an alcohol-induced disorder in substance use disorder treatment. Among cases with a substance-induced disorder in substance use disorder treatment, methamphetamine use was reported among 26.4% (95,055 / 359,486) of cases, and cannabis use was reported among 23.6% (84,743 / 359,486) of cases.

### Mental health disorder treatment cases: Mental health disorders

**Fig 5:**
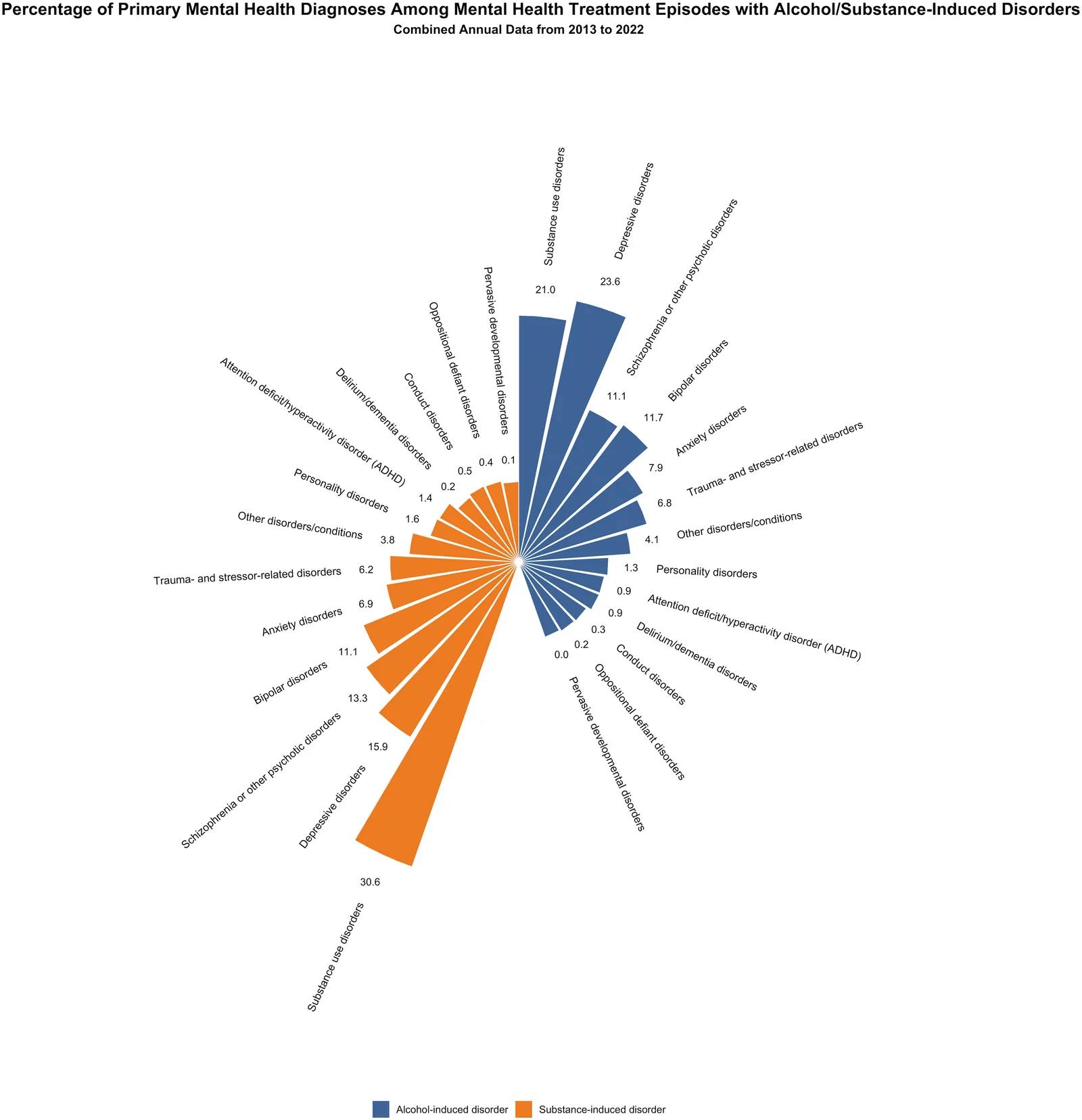
Percentage of Primary Mental Health Diagnoses Among Mental Health Treatment Episodes with Alcohol/Substance-Induced Disorders. Combined Annual Data from 2013 to 2022”.

The percentage of primary mental health disorders among cases with both alcohol and substance-induced disorders receiving treatment in the mental health disorder treatment setting may be found in Fig 5. Depressive disorders were identified as the primary mental health disorder diagnosis among 23.6% (26,417 / 111,720) of cases with an alcohol-induced disorder in mental health disorder treatment, followed by substance use disorders at 21.0% (23,433 / 111,720), and bipolar disorders at 11.7% (13,098 / 111,720). Among cases with a substance-induced disorder in mental health treatment, 30.6% (100,412 / 328,212) had a substance use disorder, followed by 15.9% (52,155 / 328,212) with a depressive disorder, and 13.3% (43,769 / 328,212) with schizophrenia or other psychotic disorders.

**Fig 6:**
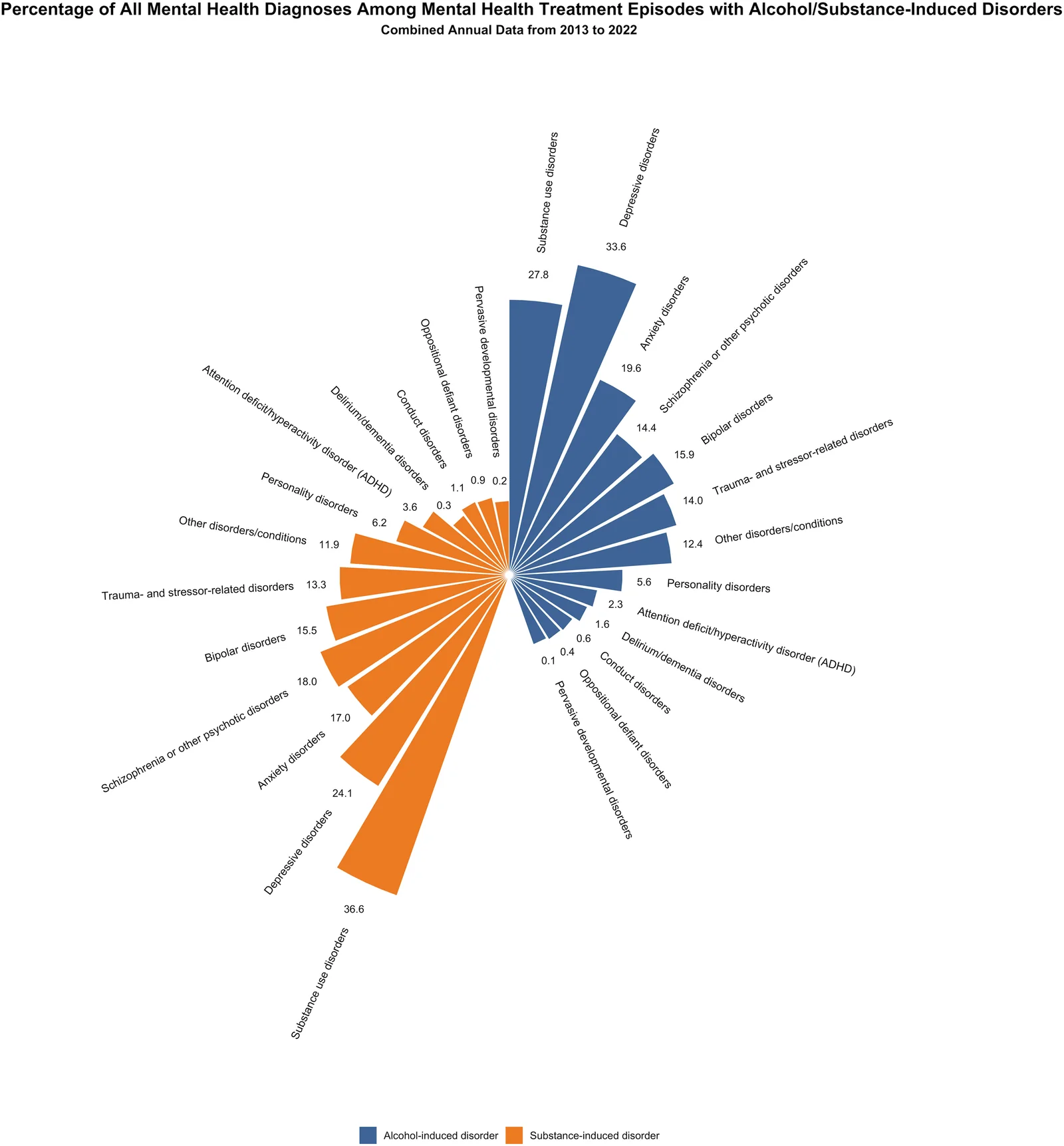
Percentage of All Mental Health Diagnoses Among Mental Health Treatment Episodes with Alcohol/Substance-Induced Disorders. Combined Annual Data from 2013 to 2022”.

As some cases also had a secondary and/or tertiary mental health disorder listed, not just a primary mental health disorder, Fig 6 shows cases with any mental health disorder reported in the mental health disorder treatment setting. Depressive disorders were identified among 33.6% (37,486 / 111,720) of cases with an alcohol-induced disorder in mental health disorder treatment, followed by substance use disorders at 27.8% (31,015 / 111,720) and anxiety disorders at 19.6% (21,870 / 111,720). Among cases with a substance-induced disorder in mental health disorder treatment, 36.6% (120, 288 / 328,212) had a substance use disorder, followed by 24.1% (79,106 / 328,212) with a depressive disorder, and 18.0% (59,228 / 328,212) with schizophrenia or other psychotic disorders.

## Discussion

From 2013 to 2022, among all mental health disorder treatment cases, those with an alcohol or substance-induced disorder made up 0.7% of cases. During the same period, alcohol or substance-induced disorders accounted for approximately 2.7% of all substance use disorder treatment cases. Additionally, the total cases involving either an alcohol or substance-induced disorder in mental health treatment or substance use disorder treatment numbered 439,932 and 459,817, respectively. Although cases with an alcohol or substance-induced disorder diagnosis represent a minority in either treatment setting, the roughly 900,000 cases meeting these criteria over the analytic period emphasize these conditions as being a public health concern [16].

Considering alcohol or substance-induced disorders include different conditions such as intoxication, withdrawal, and mental conditions resulting in anxiety, depressive, psychotic, or manic symptoms [4], there is variability in treatment needs and how these needs present in treatment. For example, people with intoxication or withdrawal symptoms are likely to require specialized substance use disorder treatment services. However, mental health symptoms can also emerge due to the experience of withdrawal, which is captured in many standardized withdrawal assessments. For example, the Clinical Institute Withdrawal Assessment for Alcohol-Revised [21], Clinical Institute Withdrawal Assessment-Benzodiazepines [22], and Subjective Opiate Withdrawal Scale [23], all include anxiety/anxious as a potential symptom. Therefore, managing both the impact of the substance and subsequent mental health symptoms can enhance the care that individuals receive [5–12]. Variability in the conditions that are induced and the severity of these substance-induced disorders are impacted by the specific substance or substances that the person uses [1].

This study examined all listed and primary substances reported among cases that received substance use disorder treatment. As might be expected, the majority (79%) of cases with an alcohol-induced disorder in substance use disorder treatment were identified as reporting alcohol use, with 68% having alcohol listed as their primary substance. Heroin and other opiates and synthetics accounted for the primary substance among 28.8% of cases with a substance-induced disorder in the substance use disorder treatment data. One study found that among 31,134 treatment episodes initiating medication for opioid use disorder, such as buprenorphine, approximately 3.5% had a substance-induced mental health disorder [24]. Further, that study found that among persons with an opioid use disorder, developing a subsequent substance-induced mental health disorder was associated with leaving treatment prematurely [24]. This highlights the importance of addressing mental health symptoms and conditions that may emerge due to substance use. Approximately 1 in 5 cases with a substance-induced disorder had methamphetamine listed as the primary substance. Methamphetamine-induced psychosis is a known mental health consequence of methamphetamine use and should be addressed in treatment to avoid persons returning to previous patterns of use [25,26]. Unlike other drug classes commonly identified in substance use treatment, such as alcohol, nicotine, opioids, and tobacco, there are no approved pharmacotherapies for methamphetamine. However, behavioral therapies such as contingency management may assist in treating a substance use disorder due to methamphetamine.[27]

This study also examined all listed and primary mental health diagnoses reported among cases that received mental health treatment. Approximately 1 in 3 cases with an alcohol-induced disorder and 1 in 4 cases with a substance-induced disorder had a depressive disorder diagnosis. When considering the primary diagnosis, approximately 1 in 4 cases with an alcohol-induced disorder and 15% of cases with a substance-induced disorder had a depressive disorder as the primary diagnosis. A review of the literature suggests that more studies are needed to distinguish substance-induced mood disorders from comorbid mood disorders in individuals with substance use disorders, as the review found inconsistent results regarding risk factors and symptoms [2]. This is an important clinical consideration since mental health disorders often co-occur with substance use disorders. However, for a mood disorder to be classified as substance-induced, substance use must be the causal factor for the condition. A study from 2012 examining 58 persons who use substances and have a mood disorder in a psychiatric unit identified that major depressive episodes were substance-induced in 72% of individuals, and more specifically, regarding the substance, 57% were alcohol-induced major depressive episodes [28].

As seen in Fig 5 and 6, bipolar disorders and schizophrenia or other psychotic disorders were in the top five of all reported mental health diagnoses and primary diagnoses. Subsequent transition from substance-induced psychosis to bipolar or schizophrenia/other psychotic disorders has been documented in the literature. The risk of transition to schizophrenia spectrum disorder is higher than the risk of transition to bipolar disorder [29]. Overall, findings from this study revealed a high level of clinical co-morbidity, with several mental health disorders showing a high prevalence among cases in the mental health treatment data.

Findings from this study suggest potential avenues for future research. First, given the often-separate expertise of mental health disorder treatment facilities and substance use disorder treatment facilities [30], studies of clinical outcomes should be conducted in both settings. Further, clinical outcomes in this sample should be examined in settings that provide only mental health or substance use disorder treatment, compared with facilities that provide integrated treatment for co-occurring mental health/substance use disorders. Future studies that can discern whether outcomes differ by the treatment facility’s specialty can provide insights into effective interventions for this clinical population. Another avenue for future research is examining the combined clinical course of the sample based on co-occurring diagnoses, such as alcohol use disorder and depressive disorder, potentially co-occurring with the induced disorder. Identifying whether the clinical course differs between those with and without a co-occurring mental health disorder when an alcohol or substance-induced disorder emerges would be a useful contribution to the literature to inform clinical care.

### Limitation

Despite employing robust statistical tools in exploring alcohol and substance-induced disorders in both mental health and substance use disorder treatments, limitations must be acknowledged. The Mental Health Client-Level Data [17] and the Treatment Episode Data Set Admissions [15] differ in units of analysis, with individuals and treatment episodes serving as the respective units. Another limitation is that the mental health treatment data includes diagnoses of mental health disorders, while the substance use disorder treatment data considers substance use as a primary, secondary, or tertiary clinical factor. This makes it difficult to consistently analyze variables across both datasets. Additionally, while alcohol-induced disorder is listed as a separate category from substance-induced disorder in these datasets, substance-induced disorder is broadly defined as conditions caused by substance use, including alcohol, based on diagnostic criteria [1]. Consequently, since this is real-world treatment data, some cases categorized as substance-induced disorders may involve alcohol as the substance. While this study provides an overall description of these cases falling under the umbrella of mental health or substance use disorder treatment, future studies are needed to examine specific levels of care such as residential treatment or outpatient treatment. Future studies examining different levels of care are needed, as outpatient treatment is typically provided to persons with less severe conditions or more stable environmental support than those in inpatient or residential treatment. Another limitation is that intoxication, withdrawal, and mental health issues are grouped under the broad diagnostic category of substance-induced disorder [1], but the datasets lack specific variables to determine which particular type of substance-induced disorder applies in each case. While this study identifies cases that have a reported alcohol- or substance-induced disorder, the true prevalence of these conditions cannot be determined in either dataset as not all people entering treatment may have been screened for these conditions. Further, the “DSMCRIT” and “SUB” variables allow only one category per case, and other categories, such as cocaine abuse, are permitted in these variables.

## Conclusion

This descriptive epidemiological study found that alcohol-induced disorder cases made up 0.2%, while substance-induced disorder cases accounted for 0.5% of the total national mental health treatment data. Substance-induced disorders are a significant public health concern, and more research is needed to better understand the clinical needs of this population [16]. Future research should focus on analyzing demographic trends of individuals with these conditions, assessing the effectiveness of treatments, identifying the most commonly used substances each year within this sample, and determining the most prevalent co-occurring mental health disorders annually among this sample.
