# Supplementary material for: Alcohol and substance-induced disorder treatment cases in mental health and addiction treatment facilities from 2013 to 2022: A descriptive study
Source: PLOS Ment Health. 2026 Sep 2;3(9):e0000706. doi: 10.1371/journal.pmen.0000706 (PMC13537537; doi:10.1371/journal.pmen.0000706)
Supplement: Supplementary file 4 [file pmen.0000706.s005.docx]

**Reviewer #3:** The manuscript is fair and I think it is good enough for publication.

**The reviewer is thanked for their initial review during the previous round, and for this feedback in response to the changes.**

**Reviewer #4:** Address
Thank you for submitting a revised manuscript. The effort is acknowledged.

**Similarly, the reviewer is thanked for their previous and current reviews, which have made this paper more robust.**

Summary
This is a descriptive study that assesses alcohol and substance-induced disorder treatment cases in mental health and addiction treatment facilities in the USA from 2013 to 2022. Secondary data was obtained from the Mental Health Client-Level Data and the Treatment Episode Data Set Admissions. Overall, the study is academically and clinically relevant. However, there are a few concerns.

General comments
• Page numbers and line numbers:
The author could have included page numbers and line numbers of corrected sections for easy review.

**This current response to reviewers lists page and line numbers to improve clarity.**

• Presentation
There are a number of statements and paragraphs that are not clear at a glance. Example:
“Although this study explores alcohol and substance-induced disorders in both mental health and substance use disorder treatments, limitations must be acknowledged.” Page 9; lines 321-322
This statement can read:
Despite employing robust statistical tools in exploring alcohol and substance-induced disorders in both mental health and substance use disorder treatments, limitations must be acknowledged.

**The previous text has been reworded based on the reviewer’s feedback as seen on page 9; line 311 of the Tracked Changes Document.**

Also, the flow and train of thoughts in some aspects of the introduction, results, discussion, limitations, and conclusion can be difficult to grasp at a glance. The author may consider involving colleagues to help with proofreading. Their suggestions may greatly enhance the flow and clarity of the presentation.

**The entire text was reviewed and edited.**

• Long sentences
I commend the effort to rewrite the long sentence cited as an example in the review comment.
“This study also examines mental health diagnoses reported in those receiving mental health treatment and substances used among individuals with an alcohol or substance-induced disorder between 2013 and 2022.” Page 3; lines 106-109
However, the reworded statement (above) could have been presented this way:
“This study also examines mental health diagnoses reported in those receiving mental health treatment. Further, it assesses substances used among individuals with an alcohol or substance-induced disorder between 2013 and 2022.”

**The previous text has been reworded based on the reviewer’s feedback as seen on page 3; line 104 of the Tracked Changes Document.**

Also, there are several long sentences, which are difficult to read. Example:
“One study analyzed the Substance Abuse and Mental Health Services Administration’s Treatment Episode Data Set Admissions [15], reviewing all cases identified as having a “substance-induced disorder” from 1992 to 2022, excluding cases with an “alcohol-induced disorder" unless examining trends of these diagnoses [16].” Page 3; lines 95-99
The statement above can read:
“A study analyzing the Substance Abuse and Mental Health Services Administration’s Treatment Episode Data Set Admissions [15], reviewed all cases identified as having a substance-induced disorder from 1992 to 2022. However, it excluded cases with an alcohol-induced disorder unless examining trends of these diagnoses [16]

**The previous text has been reworded to the following on page 3; line 93 of the Tracked Changes Document.**

**“A study analyzing the Substance Abuse and Mental Health Services Administration’s Treatment Episode Data Set Admissions [15], reviewed all cases identified as having a “substance-induced disorder” from 1992 to 2022 [16]. However, the study excluded cases with an “alcohol-induced disorder" except where trends of these diagnoses were examined [16].”**

“Another limitation is that the mental health treatment data includes diagnoses of mental health disorders, while the substance use disorder treatment data considers substance use as a primary, secondary, or tertiary clinical factor, making it difficult to analyze these aspects consistently across both datasets.”
The statement above can read:
“Another limitation is that the mental health treatment data includes diagnoses of mental health disorders, while the substance use disorder treatment data considers substance use as a primary, secondary, or tertiary clinical factor. This makes it difficult to consistently analyze both datasets.”

**The previous text has been reworded to the following on page 9; line 317 of the Tracked Changes Document.**

**“Another limitation is that the mental health treatment data includes diagnoses of mental health disorders, while the substance use disorder treatment data considers substance use as a primary, secondary, or tertiary clinical factor. This makes it difficult to consistently analyze variables across both datasets.”**

These are a few examples of the long sentences. The author was expected to go through the entire manuscript and reword the long sentences to improve the presentation.

**The entire text was reviewed and edited to reduce long sentences and improve clarity.**

Introduction
“Substance-induced disorders are a category of conditions caused by substance use (ref).” Page 3; line 75

**The reference seen below was added on page 3; line 76 of the Tracked Changes Document.**

**Substance-induced disorders are a category of conditions caused by substance use [1].**

“psychosis symptoms” Page 3; line 83
The phrase can read “psychotic symptoms”.

**This text was changed to “symptoms of psychosis on page 3; line 83 of the Tracked Changes Document.**

“However, the literature lacks descriptive studies on substance-induced disorder cases within both mental health and substance use treatment settings, alongside clinical characteristics such as substances used and mental health diagnoses.” Page 3; lines 88-91
The statement above is not clear. Please, is the highlighted phrase referring to comorbid mental health disorders? If so, the author should kindly make it clear. This can be done by rewording the statement or breaking the statement into two or three clear sentences.

**The previous text has been reworded to the following on page 3; line 88 of the Tracked Changes Document.**

**“However, the literature lacks descriptive studies on substance-induced disorder cases within both mental health and substance use treatment settings. Data on substance-induced disorder comorbidities, such as mental health and substance use disorders, are also lacking.”**

“Given the unique nature of substance-induced disorders, facilities specializing in treating mental health and/or substance use disorders may encounter individuals with these conditions. However, the literature lacks descriptive studies on substance-induced disorder cases within both mental health and substance use treatment settings, alongside clinical characteristics such as substances used and mental health diagnoses. Descriptive studies are important to the research literature as they can highlight the prevalence of specific conditions and who is affected by them.” Page 3; lines 86-93

I appreciate the effort in trying to link this paragraph to the preceding one. However, I humbly think a different opening statement will help. The facilities were designed to handle such cases, so they are expected to receive such cases.

**Not all mental health or addiction facilities are designed to handle cases with comorbidities, even those that may receive such cases, unfortunately. This opening sentence points to this unfortunate clinical reality.**

Materials and Methods
Response is well noted. Thank you.

**The reviewer is thanked for this feedback.**

Results
Response is well noted. The figures added to the reviewer response appear better. Thank you.

**The reviewer is thanked for this feedback.**

Discussion
The presentation (flow and clarity) of the discussion can be improved. For example, the second paragraph of the “Discussion” talks about the prevalence of the reported substances, if I am not mistaken. However, it is very difficult to correlate the long and winding opening statements of the paragraph to the import of the paragraph. Page 7; lines 245-258

**The second paragraph of the discussion was added in response to Reviewer #2's feedback during the second revision. While the reviewer considers the opening sentences long, the extra details were provided to highlight the complexity of these conditions called substance-induced disorders.**

Also, I acknowledge the position of the author concerning the lack of data. However, like all other novel studies, the discussion can be done in the light of other national, regional, continental, or global studies.

However, the final decision to accept the “Discussion” in this current format rests with the editor.

**The reviewer is correct; if suitable studies were available to highlight, they would certainly be included to incorporate the broader research literature. However, after a thorough review of the literature, studies using national treatment data focusing on substance-induced disorders as a whole are nascent (nationally, regionally, continentally, and globally). The only relevant study is the Fedotov and Shustov study that directly addresses this research area (**[**https://doi.org/10.2174/0118750362402771250908034339**](https://doi.org/10.2174/0118750362402771250908034339)**).**

**To further highlight this reality, this is a direct quote from the cited Fedotov and Shustov paper: “However, there is a lack of data on the trends in the incidence of substance-induced mental disorders at a general population level. These data would allow a better understanding of the impact of the general addiction factors, including the emergence of novel psychoactive substances, on this type of addiction complication.” To address this gap, the discussion section largely focused on clinical aspects of these conditions.**

Limitation
Response is well noted. Thank you.

**The reviewer is thanked for this feedback.**

Conclusion
The conclusion is long. Page 10
It is expected to be succinct. It is supposed to include a very brief background of the study, key findings, and recommendations or implications for future studies. This can be done in a few sentences. The conclusion is not supposed to be a rehash of the results (with figures).

**The conclusion was reduced to the following on page 10; line 344 of the Tracked Changes Document.**

**“This descriptive epidemiological study found that alcohol-induced disorder cases made up 0.2%, while substance-induced disorder cases accounted for 0.5% of the total national mental health treatment data. Substance-induced disorders are a significant public health concern, and more research is needed to better understand the clinical needs of this population [16]. Future research should focus on analyzing demographic trends of individuals with these conditions, assessing the effectiveness of treatments, identifying the most commonly used substances each year within this sample, and determining the most prevalent co-occurring mental health disorders annually among this sample.”**

References
Some of the issues concerning references were missed in the response. Examples:
“Substance-induced disorders are a category of conditions caused by substance use” (ref). Page 3; line 75

**The reference seen below was added on page 3; line 76 of the Tracked Changes Document.**

**“Substance-induced disorders are a category of conditions caused by substance use [1].”**

“Therefore, managing both the impact of the substance and subsequent mental health symptoms can enhance the care that individuals receive (ref). Variability in the conditions that are induced and the severity of these substance-induced disorders are impacted by the specific substance or substances that the person uses” (ref). Page 7; lines 255-258
Please, providing references for these statements will be helpful.

**The reference seen below was added on page 7; line 247 of the Tracked Changes Document.**

**“Therefore, managing both the impact of the substance and subsequent mental health symptoms can enhance the care that individuals receive [5-12]. Variability in the conditions that are induced and the severity of these substance-induced disorders are impacted by the specific substance or substances that the person uses [1].”**
