# Supplementary material for: Alcohol and substance-induced disorder treatment cases in mental health and addiction treatment facilities from 2013 to 2022: A descriptive study
Source: PLOS Ment Health. 2026 Sep 2;3(9):e0000706. doi: 10.1371/journal.pmen.0000706 (PMC13537537; doi:10.1371/journal.pmen.0000706)
Supplement: Supplementary file 5 [file pmen.0000706.s002.docx]

PMEN-D-25-00524
Alcohol and Substance-Induced Disorder Treatment Cases in Mental Health and Addiction Treatment Facilities from 2013 to 2022
PLOS Mental Health

Dear Dr. Ware,

Thank you for submitting your manuscript to PLOS Mental Health. After careful consideration, we feel that it has merit but does not fully meet PLOS Mental Health’s publication criteria as it currently stands. Therefore, we invite you to submit a revised version of the manuscript that addresses the points raised during the review process.


Please submit your revised manuscript by Jan 24 2026 11:59PM. If you will need more time than this to complete your revisions, please reply to this message or contact the journal office at [mentalhealth@plos.org](mailto:mentalhealth@plos.org). When you're ready to submit your revision, log on to <https://www.editorialmanager.com/pmen/> and select the 'Submissions Needing Revision' folder to locate your manuscript file.

Please include the following items when submitting your revised manuscript:

- A rebuttal letter that responds to each point raised by the editor and reviewer(s). You should upload this letter as a separate file labeled 'Response to Reviewers'.
- A marked-up copy of your manuscript that highlights changes made to the original version. You should upload this as a separate file labeled 'Revised Manuscript with Track Changes'.
- An unmarked version of your revised paper without tracked changes. You should upload this as a separate file labeled 'Manuscript'.

Guidelines for resubmitting your figure files are available below the reviewer comments at the end of this letter.

We look forward to receiving your revised manuscript.

Kind regards,

Gellan Karamallah Ramadan Ahmed
Academic Editor
PLOS Mental Health

Journal Requirements:

If the reviewer comments include a recommendation to cite specific previously published works, please review and evaluate these publications to determine whether they are relevant and should be cited. There is no requirement to cite these works unless the editor has indicated otherwise. 

Additional Editor Comments (if provided):


[Note: HTML markup is below. Please do not edit.]

Reviewers' comments:

Reviewer's Responses to Questions

**Comments to the Author**

1. Does this manuscript meet PLOS Mental Health’s [publication criteria](https://nam12.safelinks.protection.outlook.com/?url=https%3A%2F%2Ftrack.editorialmanager.com%2FCL0%2Fhttps%3A%252F%252Fjournals.plos.org%252Fmentalhealth%252Fs%252Fcriteria-for-publication%2F1%2F010f019b07640ad2-4dd0242a-69ec-478c-887c-ffa412b40bd8-000000%2FH32L6YUoy6GKCQlv1eghclD59Zr7ZfYNbOPI9EF923g%3D241&data=05%7C02%7C%7C9238189ba3a44dcd4fbf08de37c6abfb%7C58b3d54f16c942d3af081fcabd095666%7C1%7C0%7C639009523622331244%7CUnknown%7CTWFpbGZsb3d8eyJFbXB0eU1hcGkiOnRydWUsIlYiOiIwLjAuMDAwMCIsIlAiOiJXaW4zMiIsIkFOIjoiTWFpbCIsIldUIjoyfQ%3D%3D%7C0%7C%7C%7C&sdata=YFtPv8ISjx8iT9chq345RP1Km92975VZMmCWyhIdG58%3D&reserved=0)? Is the manuscript technically sound, and do the data support the conclusions? The manuscript must describe methodologically and ethically rigorous research with conclusions that are appropriately drawn based on the data presented.

Reviewer #1: Partly

Reviewer #2: Yes

2. Has the statistical analysis been performed appropriately and rigorously?

Reviewer #1: N/A

Reviewer #2: No

3. Have the authors made all data underlying the findings in their manuscript fully available (please refer to the Data Availability Statement at the start of the manuscript PDF file)?

The [PLOS Data policy](https://nam12.safelinks.protection.outlook.com/?url=https%3A%2F%2Ftrack.editorialmanager.com%2FCL0%2Fhttps%3A%252F%252Fjournals.plos.org%252Fmentalhealth%252Fs%252Fdata-availability%2F1%2F010f019b07640ad2-4dd0242a-69ec-478c-887c-ffa412b40bd8-000000%2FKcTKoJuc11aIunwnD6heMzUxS693JTH7MDhSpgU4d00%3D241&data=05%7C02%7C%7C9238189ba3a44dcd4fbf08de37c6abfb%7C58b3d54f16c942d3af081fcabd095666%7C1%7C0%7C639009523622356829%7CUnknown%7CTWFpbGZsb3d8eyJFbXB0eU1hcGkiOnRydWUsIlYiOiIwLjAuMDAwMCIsIlAiOiJXaW4zMiIsIkFOIjoiTWFpbCIsIldUIjoyfQ%3D%3D%7C0%7C%7C%7C&sdata=LF2vpq14i2B4HvBjGiOJJAgT0e96gdN4b%2FoQdk661iY%3D&reserved=0) requires authors to make all data underlying the findings described in their manuscript fully available without restriction, with rare exception. The data should be provided as part of the manuscript or its supporting information, or deposited to a public repository. For example, in addition to summary statistics, the data points behind means, medians and variance measures should be available. If there are restrictions on publicly sharing data—e.g. participant privacy or use of data from a third party—those must be specified.

Reviewer #1: No

Reviewer #2: Yes

4. Is the manuscript presented in an intelligible fashion and written in standard English?

PLOS Mental Health does not copyedit accepted manuscripts, so the language in submitted articles must be clear, correct, and unambiguous. Any typographical or grammatical errors should be corrected at revision, so please note any specific errors here.

Reviewer #1: Yes

Reviewer #2: Yes

5. Review Comments to the Author

Please use the space provided to explain your answers to the questions above. You may also include additional comments for the author, including concerns about dual publication, research ethics, or publication ethics. (Please upload your review as an attachment if it exceeds 20,000 characters)

**The editors and reviewers are thanked for their suggestions, which have improved the paper, and for the opportunity to resubmit it. Please find specific responses to the items below.**

Reviewer #1: This manuscript provides a national-level analysis of alcohol-induced and substance-induced disorder diagnoses across U.S. mental health and substance use disorder (SUD) treatment systems from 2013–2022. The manuscript is generally well written, methodologically straightforward, and supported by appropriate data sources. However, it would benefit from stronger framing, greater methodological clarity, more detailed analysis of trends, and a more nuanced discussion connecting the results to existing literature. Several limitations also need more explicit and specific treatment. Overall, I recommend major revisions to strengthen interpretation, clarity, and methodological transparency.

**I thank Reviewer 1 for their expertise, feedback, and review. To improve clarity, the following statement about the aims was added to the introduction section:**

**“Three aims guided this research study: [a] Identify the percentage of cases with alcohol-induced disorders or substance-induced disorders in mental health and substance use disorder treatment settings, [b.] Examine the prevalence of other mental health diagnoses, such as anxiety and depressive disorders, among cases with alcohol-induced disorders or substance-induced disorders in mental health treatment, and [c.] Examine the prevalence of substances used, such as alcohol and cannabis, among cases with alcohol-induced disorders or substance-induced disorders in mental health treatment.”**

**The following details were added to the methods section to describe the sample selection variable, which improves methodological interpretation and enables direct replication of the study. This will permit anyone to be able to select the sample and completely replicate the study.**

**“The variable in the Mental Health Client-Level Data is “SUB”. The variable in the Treatment Episode Data Set Admissions is “DSMCRIT.””**

The aim of the article is not clearly stated in the abstract, and emphasizing it more explicitly would help readers understand the purpose and contribution of the study. Additionally, although the paper claims to analyze trends, it does not clearly describe how the reported values change over time. The revised manuscript should be consistent—either functioning as a high-level summary or offering an account of periodical change.

**The following sentence was added to the abstract to describe the study’s aim.**

**“The primary aim of this study was to identify the percentage of cases with alcohol-induced disorders or substance-induced disorders in mental health and substance use disorder treatment settings.”**

**Furthermore, all references to "trend" or "trends" in this study were removed to maintain a descriptive focus.**

I also noted that Table 1 does not appear to be referenced in the text. The figures are difficult to read and appear pixelated; higher-resolution images would improve readability.

**In the initial submission and in this revision, the following wording may be found:**

**“Demographic characteristics such as age, gender, and race and ethnicity may be found in Table 1.”**

**The figures were exported from R as high resolution figures. For this revision, they were then uploaded and marked as “Fixed” by the PLOS’s NAAS tool link that was provided by PLOS at the end of this revision. However, the figures still appear slightly pixilated. Yet when I clicked the “Click here to access/download; Figure” in the upper-right corner of the submission proof, I could see the complete figure, un-pixelated as uploaded. I am unsure of how to fix this since the downloadable figure appears to be correct, the figure was created as high resolution in R, and the figures were identified as fixed by the PLOS figure tool. Below is a screenshot of the PLOS figure tool that was used to fix the figures.**

**However, for full transparency, I want to ensure the Reviewers can view the figures. Please find these at the end of this reviewer response document.**

Regarding lines 309–310, the statement about further research is broad and therefore not very informative. The authors should be more precise about what types of future research are needed. For example, it would be helpful to specify what kinds of studies could translate these descriptive findings into actionable steps to improve interventions and enhance care for this population.

**By focusing on different potential treatment outcome comparisons between both settings, the following wording was added in the discussion section:**

**“Findings from this study suggest potential avenues for future research. First, given the often-separate expertise of mental health disorder treatment facilities and substance use disorder treatment facilities, studies of clinical outcomes should be conducted in both settings. Further, clinical outcomes in this sample should be examined in settings that provide only mental health or substance use disorder treatment, compared with facilities that provide integrated treatment for co-occurring mental health/substance use disorders. Another avenue for future research is examining the combined clinical course of the sample based on co-occurring diagnoses, such as alcohol use disorder and depressive disorder, potentially co-occurring with the induced disorder.”**

Reviewer #2: Thank you very much for the opportunity to review this interesting article. The subject matter discussed would be, I believe, of interest to the readership of PLOS Mental Health. However, my main concern centres around the analysis of what is a very large dataset, which has the potential to provide significant insight. The study only considers univariate statistics in the analysis, while it would be useful to see, as you say, “national trends of alcohol or substance-induced disorders in the mental health treatment setting alongside substance use disorder cases”. Multivariate analysis would be welcomed here, and I would advise discussing with a statistician the best approach to take to conduct robust analysis. This should lead to a significant revision and strengthening of the discussion section, in which I would advise that you think much more carefully about what the results are and are not showing you, and the potential implications of this.
In summary, while the paper has the potential to offer important insights in this area, more robust analysis and discussion is first required.

**I thank Reviewer 2 for their expertise, feedback, and review. The reviewer is correct; there are several untested analyses. Admittedly, this was purposeful, to focus on a descriptive paper. By introducing this novel study, which examines a national sample of individuals with either alcohol or substance induced disorder in a national sample across two large treatment settings in the United States, this paper will generate future ideas for researchers. For example, other researchers with expertise in demography may see this initial paper and conduct future studies on the regional and demographic characteristics. Essentially, future researchers could conduct hundreds of different analyses based on the current datasets, which is largely unexamined in the addiction and mental health research space.**

**By introducing this dataset and highlighting the potential for examining these induced disorders, this researcher hopes that this paper, which focuses solely on descriptive analysis, will point future researchers to different research avenues. Following the “Framework for Descriptive Epidemiology” described by Lesko et al. (2022;** [**https://doi.org/10.1093/aje/kwac115**](https://doi.org/10.1093/aje/kwac115)**), this paper was explicitly described as a descriptive study.**

**Univariable statistics in a descriptive epidemiological study also make useful contributions to the literature, especially when the focus is on a sample largely absent from the research literature. More was done in this revision to identify this paper as a descriptive study, including adding the following wording to the title: “A Descriptive Study”**

**Furthermore, all references to "trend" or "trends" in this study were removed to maintain a descriptive focus.**

6. PLOS authors have the option to publish the peer review history of their article ([what does this mean?](https://nam12.safelinks.protection.outlook.com/?url=https%3A%2F%2Ftrack.editorialmanager.com%2FCL0%2Fhttps%3A%252F%252Fjournals.plos.org%252Fmentalthealth%252Fs%252Feditorial-and-peer-review-process%2523loc-peer-review-history%2F1%2F010f019b07640ad2-4dd0242a-69ec-478c-887c-ffa412b40bd8-000000%2FoejG9627SvsLrJh64Ynkk6GXO0FO0qGY7YIDuQV4pKs%3D241&data=05%7C02%7C%7C9238189ba3a44dcd4fbf08de37c6abfb%7C58b3d54f16c942d3af081fcabd095666%7C1%7C0%7C639009523622376259%7CUnknown%7CTWFpbGZsb3d8eyJFbXB0eU1hcGkiOnRydWUsIlYiOiIwLjAuMDAwMCIsIlAiOiJXaW4zMiIsIkFOIjoiTWFpbCIsIldUIjoyfQ%3D%3D%7C0%7C%7C%7C&sdata=mMdr%2Ba8mhl2MOkaAkrxM7iEEewz6fJSQ8cKn1XxfB5A%3D&reserved=0)). If published, this will include your full peer review and any attached files.


**Do you want your identity to be public for this peer review?** If you choose “no”, your identity will remain anonymous but your review may still be made public.


For information about this choice, including consent withdrawal, please see our [Privacy Policy](https://nam12.safelinks.protection.outlook.com/?url=https%3A%2F%2Ftrack.editorialmanager.com%2FCL0%2Fhttps%3A%252F%252Fwww.plos.org%252Fprivacy-policy%2F1%2F010f019b07640ad2-4dd0242a-69ec-478c-887c-ffa412b40bd8-000000%2F9LR93Vb1mfcGXxBbCwOZaBxecxsKJawJZXVO9H0WvoQ%3D241&data=05%7C02%7C%7C9238189ba3a44dcd4fbf08de37c6abfb%7C58b3d54f16c942d3af081fcabd095666%7C1%7C0%7C639009523622396718%7CUnknown%7CTWFpbGZsb3d8eyJFbXB0eU1hcGkiOnRydWUsIlYiOiIwLjAuMDAwMCIsIlAiOiJXaW4zMiIsIkFOIjoiTWFpbCIsIldUIjoyfQ%3D%3D%7C0%7C%7C%7C&sdata=%2FQw24BAAs6oEd%2FaIm65Ez%2FDceMuN7WCy449xjlUTH20%3D&reserved=0).

Reviewer #1: No

Reviewer #2: No

[NOTE: If reviewer comments were submitted as an attachment file, they will be attached to this email and accessible via the submission site. Please log into your account, locate the manuscript record, and check for the action link "View Attachments". If this link does not appear, there are no attachment files.]

Figure Resubmissions:

While revising your submission, we strongly recommend that you use PLOS’s NAAS tool (<https://ngplosjournals.pagemajik.ai/artanalysis>) to test your figure files. NAAS can convert your figure files to the TIFF file type and meet basic requirements (such as print size, resolution), or provide you with a report on issues that do not meet our requirements and that NAAS cannot fix.

After uploading your figures to PLOS’s NAAS tool - <https://ngplosjournals.pagemajik.ai/artanalysis>, NAAS will process the files provided and display the results in the "Uploaded Files" section of the page as the processing is complete. If the uploaded figures meet our requirements (or NAAS is able to fix the files to meet our requirements), the figure will be marked as "fixed" above. If NAAS is unable to fix the files, a red "failed" label will appear above. When NAAS has confirmed that the figure files meet our requirements, please download the file via the download option, and include these NAAS processed figure files when submitting your revised manuscript.
