# Supplementary material for: Alcohol and substance-induced disorder treatment cases in mental health and addiction treatment facilities from 2013 to 2022: A descriptive study
Source: PLOS Ment Health. 2026 Sep 2;3(9):e0000706. doi: 10.1371/journal.pmen.0000706 (PMC13537537; doi:10.1371/journal.pmen.0000706)
Supplement: Supplementary file 6 [file pmen.0000706.s003.docx]

**The editors and reviewers are thanked for the opportunity to resubmit this paper for publication consideration. Please find responses to specific items below.**

**Note to the Editors:**

**Although I used the PLOS PACE tool, the figures were still blurry on the final proof. Please also find the figures below.**

Reviewer #1: My concerns have been adressed.

**Reviewer 1 is thanked for their review of this manuscript.**

Reviewer #2: Thank you for the submission of your revised article. You have clarified that as this is a descriptive study, no further analysis will be done. As per Lesko et al. (2022) <https://doi.org/10.1093/aje/kwac115> which has guided your study, further detail is still required as to the action(s) that might be informed by the results that you have found.
Your interpretation also requires greater depth, specifically regarding how the aforementioned results could inform or improve future practice and outcomes and the future research that is required for this to be feasible. For example, why would it be important for future research to examine inpatient compared to outpatient data? What would the clinical significance be to this?

**Reviewer 2 is thanked for this great suggestion. The paper has now been expanded to point to potential future actions in real-world treatment settings, which expanded the Discussion section to slightly over 1,000 words. The discussion and limitations sections has been expanded to include the following:**

**“Considering alcohol or substance-induced disorders include different conditions such as intoxication, withdrawal, and mental conditions resulting in anxiety, depressive, psychotic, or manic symptoms, there is variability in treatment needs and how these needs present in treatment. For example, persons presenting to treatment with intoxication or withdrawal symptoms are likely to require specialized substance use disorder treatment services. However, mental health symptoms can also emerge due to the experience of withdrawal, which is captured in many standardized withdrawal assessments. For example, the Clinical Institute Withdrawal Assessment for Alcohol-Revised, Clinical Institute Withdrawal Assessment-Benzodiazepines, and Subjective Opiate Withdrawal Scale [23], all include anxiety/anxious as a potential symptom. Therefore, managing both the impact of the substance and subsequent mental health symptoms can enhance the care that individuals receive. Variability in the conditions that are induced and the severity of these substance-induced disorders are impacted by the specific substance or substances that the person uses.”**

**“Future studies that can discern whether outcomes differ by the treatment facility's specialty can provide insights into effective interventions for this clinical population. ”**

**“Identifying whether the clinical course differs between those with and without a co-occurring mental health disorder when an alcohol or substance-induced disorder emerges would be a useful contribution to the literature to inform clinical care.”**

**“Future studies examining different levels of care are needed, as outpatient treatment is typically provided to persons with less severe conditions or more stable environmental support than those in inpatient or residential treatment. Therefore, these different levels of care may often represent different client populations receiving services.”**

**“While this study identifies cases flagged as having an alcohol- or substance-induced disorder, the true prevalence of these conditions cannot be determined in either dataset. This is because the “DSMCRIT” and “SUB” variables allow only one category per case, and other categories, such as cocaine abuse, are permitted in these variables.”**

**Note to the Editors:**

**Although I used the PLOS PACE tool, the figures were still blurry on the final proof. Please find the figures here as well.**
