# Supplementary material for: Alcohol and substance-induced disorder treatment cases in mental health and addiction treatment facilities from 2013 to 2022: A descriptive study
Source: PLOS Ment Health. 2026 Sep 2;3(9):e0000706. doi: 10.1371/journal.pmen.0000706 (PMC13537537; doi:10.1371/journal.pmen.0000706)
Supplement: Supplementary file 7 [file pmen.0000706.s004.docx]

Please use the space provided to explain your answers to the questions above. You may also include additional comments for the author, including concerns about dual publication, research ethics, or publication ethics. (Please upload your review as an attachment if it exceeds 20,000 characters)

**The editors and reviewers are thanked for the opportunity to resubmit this paper. Thank you for your expert review and feedback. Please find responses to specific items below:**

**Reviewer #3:** Lines 89 to 93: ‘Due to this gap, one study analyzed the Substance 90 Abuse and Mental Health Services Administration’s Treatment Episode Data Set 91 Admissions [15], reviewing all cases identified as having a “substance-induced disorder” 92 from 1992 to 2022, excluding cases with an “alcohol-induced disorder" unless 93 examining trends of these diagnoses [16]’. The beginning of this argument ‘Due to this gap’ is weak. Kindly rephrase.

**The beginning of the sentence was removed. Instead, now the sentence reads as the following:**

**“One study analyzed the Substance Abuse and Mental Health Services Administration’s Treatment Episode Data Set Admissions, reviewing all cases identified as having a “substance-induced disorder” from 1992 to 2022, excluding cases with an “alcohol-induced disorder" unless examining trends of these diagnoses.”**

Lines 104- 111: ‘[a] Identify the percentage of cases flagged as having 105 alcohol-induced disorders or substance-induced disorders in mental health and 106 substance use disorder treatment settings, [b.] Examine the prevalence of other mental 107 health diagnoses, such as anxiety and depressive disorders, among cases with alcohol108 induced disorders or substance-induced disorders in mental health treatment, and [c.] 109 Examine the prevalence of substances used, such as alcohol and cannabis, among 110 cases with alcohol-induced disorders or substance-induced disorders in mental health 111 treatment’. You do not need to enumerate your objectives. I will advise that you report the main study objective and other specific study objectives can be discussed in the ‘Discussion section’.

**The aims for the study were added to the introduction based on feedback from Reviewer 1 during revision 1 of 3.**

**Further, the motivation and rationale for stating the complete study objectives are to provide clarity, as per the STROBE Statement checklist of items that should be included in reports of observational studies (https://www.equator-network.org/reporting-guidelines/strobe/). To provide clarity, STROBE guidelines suggest including the following in the introduction section: “State specific objectives, including any prespecified hypotheses.” Adding these details at the front of the paper can provide clarity for potential readers. The STROBE statement suggests also including the following in the discussion section, “Summarize key results with reference to study objectives.” While this can certainly add redundancy as the reviewer highlights, it also improves overall clarity to introduce the objectives in the introduction and return to them in the discussion.**

Lines 225-228: ‘Building upon the research by Fedotov and Shustov [16], which examined substance226 induced disorder treatment in the addiction setting, this is the first study to analyze 227 alcohol-induced and substance-induced disorders in national treatment records for 228 mentalhealth and substance use issues in the United States’. This is not necessary here. The statement was mentioned earlier in the Introduction section (lines 95- 100). The first paragraph of your discussion section should report the main findings of your study.

**The sentence that the reviewer described at the beginning of the discussion was removed for this revision.**

Lines 343- 359: ‘Conclusion’. Your conclusion is weak. You do not have to repeat your results in the conclusion. You are only to provide your readers with a summary of the interesting findings or novel findings of your research, not necessarily repeating figures from the results. Recommend in one or two succinct paragraphs and maybe make a key recommendation for future research.

**The conclusions section has been reduced to approximately seven sentences that highlight the primary study findings and future research. However, details regarding the most common mental health disorders and implicated substances were added to the conclusion per Reviewer 4’s feedback.**

**Reviewer #4:** Address
Thank you for submitting a revised manuscript.

Summary
This is a descriptive study that assesses alcohol and substance-induced disorder treatment cases in mental health and addiction treatment facilities in the USA from 2013 to 2022. Secondary data was obtained from the Mental Health Client-Level Data and the Treatment Episode Data Set Admissions. Overall, the study is academically and clinically relevant. However, there are a few concerns.

General comments
The term “flagged”, though correct, should be reconsidered. Terms such as “identified”, implicated, etc., are more suitable for a study of this nature.

**All four mentions of the word “flagged” were removed**

Also, there are a few grammatical errors that require the author’s attention. Example:
“For example, persons presenting to treatment…” Page 7; line 242
The author should kindly go through the entire manuscript and make the necessary corrections.

**This sentence was changed to the following: “For example, people with intoxication or withdrawal symptoms are likely to require specialized substance use disorder treatment services.”**

Furthermore, some of the sentences are quite long and difficult to read. Example:
“To further explore clinical diagnostic considerations, this study also examines mental healthdiagnoses reported in those receiving mental health treatment and substances used among individuals with an alcohol or substance-induced disorder who received treatment in either setting between 2013 and 2022.” Page 3; lines 100-103
The long sentences should be made shorter, if possible.

**This sentence was reworded to the following:**

**“This study also examines mental health diagnoses reported in those receiving mental health treatment and substances used among individuals with an alcohol or substance-induced disorder between 2013 and 2022.”**

Finally, it will be insightful to know the following trends:
a. Mental disorder with the highest prevalence for each year (between 2013 and 2022).
b. The age group with the highest prevalence of “substance-induced mental disorder” and “alcohol-induced mental disorder” for each year (between 2013 and 2022).
c. Most reported substances for each year (between 2013 and 2022).
d. Sex and race distribution for a, b, and c for each year (between 2013 and 2022).
This will inform policy-makers whether the implicated age group, mental disorders, and substances with the highest prevalence are changing over the years, and advise the design of further studies and interventions.
The author can ignore the final point if it is out of the study’s scope.

**The reviewer highlights excellent future research questions. These will be included in follow-up studies. Essentially, each one of these points is robust enough to warrant its own paper. These details were added to the conclusion section as areas of future research.**

**“Future research should focus on analyzing demographic trends of individuals with these conditions, assessing the effectiveness of treatments, identifying the most commonly used substances each year within this sample, and determining the most prevalent co-occurring mental health disorders annually among this sample.”**

Abstract
The abstract provides a sufficient summary of the background, methods, results, and conclusion of the study.

**The reviewer is thanked for this feedback.**

Introduction
“The mental health symptoms resulting from substance-induced disorders are particularly unique, as clinicians must address both the emerging psychological issues and the underlying substance use. In fact, individuals with substance-induced mental health symptoms, such as substance-induced psychosis [5-12], require treatment that considers both mental health and substance use concerns.” Page 3; lines 78-83
The highlighted statement appears to give clarity to the preceding statement. However, the terms and construct make it look similar to the preceding statement. The author can cite specific disorders to better clarify the preceding statement. See below:
“The mental health symptoms resulting from substance-induced disorders are particularly unique, as clinicians are required to address both the emerging psychological issues and the underlying substance use. For example, in the management of alcohol-induced psychotic disorder, it is recommended that the treatment address the psychotic disorder and alcohol use concern.”
Or
“The mental health symptoms resulting from substance-induced disorders are particularly unique, as clinicians are required to address both the emerging psychological issues and the underlying substance use. For example, in the management of cannabis-induced psychotic disorder, it is recommended that the treatment address the psychotic disorder and the concern of cannabis use.”

**The sentences have been reworded to the following:**

**“The mental health symptoms resulting from substance-induced disorders are particularly unique, as clinicians must address both the emerging psychological issues and the underlying substance use. For example, in the management of substance-induced psychosis, it is recommended that the treatment address the psychosis symptoms and the substance(s) of concern.”**

“Given the unique nature of substance-induced disorders, it is reasonable that facilities specializing in treating mental health disorders, substance use disorders, or both may encounter individuals with these conditions. However, the literature lacks descriptive studies [13, 14] on substance-induced disorder cases within both mental health and substance use treatment settings, alongside clinical characteristics such as substances used and mental health diagnoses.” Page 3; lines 84-89
I humbly submit that the statement above can be improved. I appreciate the intent of the author in trying to link the substance-induced mental disorders with the settings in which they are managed. However, the construction makes it difficult to get the correlation. Also, the succeeding sentence on descriptive studies can be re-examined. The author should kindly consider the construction of the statement above to improve the flow between the sentences and to clearly indicate the import of the statement. Finally, references 13 and 14 talk about the framework for descriptive epidemiology and heart failure epidemiology, respectively. The context of the limited descriptive studies mentioned in those papers, in my humble opinion, is related to their subject of study. While I acknowledge that references 13 and 14 support the author’s argument that there is a (general) lack of descriptive studies, it will be more appropriate to demonstrate, specifically, that there is a lack of descriptive studies on alcohol and substance-induced disorder treatment cases in mental health and addiction treatment facilities. This will further justify the current study.

**The sentences have been reworded to the following:**

**“Given the unique nature of substance-induced disorders, facilities specializing in treating mental health and/or substance use disorders may encounter individuals with these conditions. However, the literature lacks descriptive studies on substance-induced disorder cases within both mental health and substance use treatment settings, alongside clinical characteristics such as substances used and mental health diagnoses. Descriptive studies are important to the research literature as they can highlight the prevalence of specific conditions and who is affected by them.”**

Materials and Methods
Efforts to access data were unsuccessful. The final decision concerning the validity of the findings, based on the data, rests with the editor.

**The exact links to the datasets were provided in the reference list and the data availability statement. As of April 30, 2026, I was to download the dataset. Both datasets are provided by the Substance Abuse and Mental Health Services Administration. Please find the Data Availability Statement below that contain the links:**

**“This study used the Substance Abuse and Mental Health Service Administration’s Mental Health Client-Level Data and the Treatment Episode Data Set Admissions. Both datasets are de-identified and publicly available. The datasets may be found using these links: [1] https://www.samhsa.gov/data/data-we-collect/mh-cld-mental-health-client-level-data/datafiles and [2] https://www.samhsa.gov/data/data-we-collect/teds-treatment-episode-data-set.”**

Results
• Figures
Some of the figures, as acknowledged by the author, are blurry and difficult to read. The author may consider alternative ways to improve the visuals, if possible.

**The PLOS figure tool was used, but the figures still appear blurry. The figures were added to this reviewer response sheet and embedded into the paper to provide clear versions of them.**

Discussion
The author reports on the prevalence of Mental Health and Substance Use Disorder Treatment Cases, Substances Used, and Mental Health Disorders under “Results”. However, it is difficult to link the author’s interpretation and reasons for the observed findings to the reported results. The author should please dwell on available studies to overtly explain why the observed “prevalences” of the alcohol-induced disorder, substance-induced disorder, implicated mental disorders, and specific substances were noticed in this current study. This can be done in the light of other national, regional, continental, or global studies.

**This is part of the novelty of this study because, with the exception of** **Fedotov and Shustov's previous work (reference provided immediately below), there is limited data on available studies examining substance-induced disorders in either setting.**

**Some studies describe specific conditions such as substance-induced psychosis or withdrawal in small samples, however, data is lacking on studies examining substance-induced disorders. The nearly 1,000-word discussion section of this current study highlights several important factors in the clinical context when considering substance-induced disorders, such as: [1] variability of substance-induced disorders, [2] withdrawal, [3] substances used, [4] mental health disorders, and [5] areas for future research. Much of the current discussion section was expanded in this way based on the previous two revisions.**

**Reference:**

**Fedotov I, Shustov D. Three Waves of Substance-Induced Mental Disorders over the Past 30 Years: Results from a Retrospective Observational Cohort Analysis. The Open Bioinformatics Journal. 2025;18. doi: doi:10.2174/0118750362402771250908034339.**

Limitation
“Future studies examining different levels of care are needed, as outpatient treatment is typically provided to persons with less severe conditions or more stable environmental support than those in inpatient or residential treatment. Therefore, these different levels of care may often represent different client populations receiving services.” Page 9; lines 330-334
In my humble opinion, the highlighted statement appears redundant.

**These sentences were initially added to provide a response to Reviewer 2 during revision 2 of 3. The second sentence was removed and now only reads as the following:**

**“Future studies examining different levels of care are needed, as outpatient treatment is typically provided to persons with less severe conditions or more stable environmental support than those in inpatient or residential treatment.”**

“While this study identifies cases flagged as having an alcohol- or substance-induced disorder, the true prevalence of these conditions cannot be determined in either dataset.” Page 9; lines 338-340.
While the sincerity of the author, with respect to the limitations of the study, is acknowledged, the inability to identify the true prevalence of the parameters being assessed raises some concern. This is because the unanswered gap (prevalence) is part of the aims of the study.

**The reviewer is correct; this limitation mainly addresses the common issue in epidemiological studies, where estimating the true prevalence of a condition in a population is difficult unless everyone is screened. Even then, the measurement's type, specificity, and sensitivity can influence prevalence results. The true prevalence of any condition in a population is very rare, even in the most rigorous studies. Therefore, this was highlighted as a standard limitation, similar to self-report bias in survey-based studies.**

Conclusion
It will be helpful to include the findings of the common mental health disorders and implicated substances identified in the study as part of the conclusion. This is because these parameters are part of the aim.

**The following sentences were added to the conclusion:**

**“In substance use disorder treatment, alcohol was the primary substance in 68% of cases with an alcohol-induced disorder, and methamphetamine was the main substance in 19% of cases with a substance-induced disorder. In mental health disorder treatment, depressive disorders were the primary mental health diagnosis among 24% of cases with an alcohol-induced disorder, and 31% of cases with a substance-induced disorder had a substance use disorder.”**
