# Supplementary material for: Alcohol and substance-induced disorder treatment cases in mental health and addiction treatment facilities from 2013 to 2022: A descriptive study
Source: PLOS Ment Health. 2026 Sep 2;3(9):e0000706. doi: 10.1371/journal.pmen.0000706 (PMC13537537; doi:10.1371/journal.pmen.0000706)
Supplement: Supplementary file 8 [file pmen.0000706.s006.docx]

**The editors and reviewers are thanked for the opportunity to strengthen and resubmit this paper. Please find responses to specific items below.**

**Reviewer #4:** Address
Thank you for submitting a revised manuscript. The effort is appreciated.

**The reviewer is thanked for the opportunity to improve this paper with your feedback.**

General comments
The comments concerning the long sentences, the structure of the conclusion, and unreferenced statements have been fairly addressed. However, there are a few comments

Introduction
“Given the unique nature of substance-induced disorders, facilities specializing in treating mental healthand/or substance use disorders may encounter individuals with these conditions. However, the literature lacks descriptive studies on substance-induced disorder cases within both mental health and substance use treatment settings.” Page 3; lines 86-89

**The text was reworded to the following on Page 3, Line 83: “Facilities that treat mental health or substance use disorders may encounter patients with substance-induced disorders. This occurs because these conditions can show symptoms of both mental health and substance use disorders. However, the literature lacks descriptive studies on substance-induced disorder cases in both mental health and substance use disorder treatment settings.”**

Previous Comment by Reviewer
I appreciate the effort in trying to link this paragraph to the preceding one. However, I humbly think a different opening statement will help. The facilities were designed to handle such cases, so they are expected to receive such cases.

Author’s Response
“Not all mental health or addiction facilities are designed to handle cases with comorbidities, even those that may receive such cases, unfortunately. This opening sentence points to this unfortunate clinical reality.”

Reviewer’s Comment
It appears the author’s response to the comment may be missing some words. It is not clear. However, the statement can be accepted since it does not alter the import of the study.

**Apologies for the confusion. I will provide a more direct response to the previous comment.**

**Some facilities focus on mental health treatment. Other facilities focus on substance use disorder treatment. Some facilities that focus on mental health treatment are not equipped to treat people with substance use disorders. Some facilities that focus on substance use disorder treatment are not equipped to treat people with mental health disorders. The behavioral health system is bifurcated. Therefore, some facilities are not inherently equipped to treat people experiencing both mental health and substance use disorder symptoms.**

**This reference highlights the bifurcated nature of the behavioral system:**

**Author: Spivak et al.,**

**Year: 2020**

**Title: Integrated dual diagnosis treatment among United States mental health treatment facilities: 2010 to 2018;**

**doi: https://doi.org/10.1016/j.drugalcdep.2020.108074**

Discussion
The concerns of the discussion have not been addressed. The final decision to accept the “Discussion” in this current format rests with the editor.

**I will include the discussion section details from the last revision below, as the responses remain accurate. The reviewer's feedback mainly focuses on two points: [1] altering paragraph 2 and [2] expanding the discussion to include national, regional, continental, or global studies.**

**Second paragraph: The entire second paragraph was added specifically to address Reviewer 2 during the second revision. Before Reviewer 2’s feedback, this paragraph did not exist. The added details in the sentence are included to fully explain these conditions. This creates a dilemma because edits were made based on one reviewer's (Reviewer 2) suggestion, while another reviewer (Reviewer 4) recommends changing this section.**

**Expanding the discussion with other studies: This study is novel, and aside from the Fedotov and Shustov study, research on the overarching diagnostic grouping of substance-induced disorders in mental health or treatment settings is limited. Considering this, other pertinent details regarding clinical care were described in the discussion section**.

**Previous Discussion section feedback here:**

**Reviewer 4’s Feedback from the previous Revision**
Discussion
The presentation (flow and clarity) of the discussion can be improved. For example, the second paragraph of the “Discussion” talks about the prevalence of the reported substances, if I am not mistaken. However, it is very difficult to correlate the long and winding opening statements of the paragraph to the import of the paragraph. Page 7; lines 245-258

**Author Response from the previous Revision**

**The second paragraph of the discussion was added in response to Reviewer #2's feedback during the second revision. While the reviewer considers the opening sentences long, the extra details were provided to highlight the complexity of these conditions called substance-induced disorders.**

**Reviewer 4 Feedback from the previous Revision**
Also, I acknowledge the position of the author concerning the lack of data. However, like all other novel studies, the discussion can be done in the light of other national, regional, continental, or global studies.

However, the final decision to accept the “Discussion” in this current format rests with the editor.

**Author Response from the previous Revision**

**The reviewer is correct; if suitable studies were available to highlight, they would certainly be included to incorporate the broader research literature. However, after a thorough review of the literature, studies using national treatment data focusing on substance-induced disorders as a whole are nascent (nationally, regionally, continentally, and globally). The only relevant study is the Fedotov and Shustov study that directly addresses this research area (**[**https://doi.org/10.2174/0118750362402771250908034339**](https://doi.org/10.2174/0118750362402771250908034339)**).**

**To further highlight this reality, this is a direct quote from the cited** **Fedotov and Shustov paper: “However, there is a lack of data on the trends in the incidence of substance-induced mental disorders at a general population level. These data would allow a better understanding of the impact of the general addiction factors, including the emergence of novel psychoactive substances, on this type of addiction complication.” To address this gap, the discussion section largely focused on clinical aspects of these conditions.**
