# Supplementary material for: Alcohol and substance-induced disorder treatment cases in mental health and addiction treatment facilities from 2013 to 2022: A descriptive study
Source: PLOS Ment Health. 2026 Sep 2;3(9):e0000706. doi: 10.1371/journal.pmen.0000706 (PMC13537537; doi:10.1371/journal.pmen.0000706)
Supplement: Peer review history

# Peer review: Decision Letter 0

**DOI:** 10.1371/journal.pmen.0000706.r001

**Authors:** Gellan Karamallah Ramadan Ahmed

10 Dec 2025

PMEN-D-25-00524

Alcohol and Substance-Induced Disorder Treatment Cases in Mental Health and Addiction Treatment Facilities from 2013 to 2022

PLOS Mental Health

Dear Dr. Ware,

Thank you for submitting your manuscript to PLOS Mental Health. After careful consideration, we feel that it has merit but does not fully meet PLOS Mental Health’s publication criteria as it currently stands. Therefore, we invite you to submit a revised version of the manuscript that addresses the points raised during the review process.

Please submit your revised manuscript by Jan 24 2026 11:59PM. If you will need more time than this to complete your revisions, please reply to this message or contact the journal office at mentalhealth@plos.org. When you're ready to submit your revision, log on to https://www.editorialmanager.com/pmen/ and select the 'Submissions Needing Revision' folder to locate your manuscript file.

Please include the following items when submitting your revised manuscript:

- A rebuttal letter that responds to each point raised by the editor and reviewer(s). You should upload this letter as a separate file labeled 'Response to Reviewers'.
- A marked-up copy of your manuscript that highlights changes made to the original version. You should upload this as a separate file labeled 'Revised Manuscript with Track Changes'.
- An unmarked version of your revised paper without tracked changes. You should upload this as a separate file labeled 'Manuscript'.

Guidelines for resubmitting your figure files are available below the reviewer comments at the end of this letter.

We look forward to receiving your revised manuscript.

Kind regards,

Gellan Karamallah Ramadan Ahmed

Academic Editor

PLOS Mental Health

Journal Requirements:

If the reviewer comments include a recommendation to cite specific previously published works, please review and evaluate these publications to determine whether they are relevant and should be cited. There is no requirement to cite these works unless the editor has indicated otherwise.

Additional Editor Comments (if provided):

[Note: HTML markup is below. Please do not edit.]

Reviewers' comments:

Reviewer's Responses to Questions

**Comments to the Author**

1. Does this manuscript meet PLOS Mental Health’s publication criteria?

Reviewer #1: Partly

Reviewer #2: Yes

**********

2. Has the statistical analysis been performed appropriately and rigorously?

Reviewer #1: N/A

Reviewer #2: No

**********

3. Have the authors made all data underlying the findings in their manuscript fully available (please refer to the Data Availability Statement at the start of the manuscript PDF file)?

The PLOS Data policy

Reviewer #1: No

Reviewer #2: Yes

**********

4. Is the manuscript presented in an intelligible fashion and written in standard English?

Reviewer #1: Yes

Reviewer #2: Yes

**********

Reviewer #1: This manuscript provides a national-level analysis of alcohol-induced and substance-induced disorder diagnoses across U.S. mental health and substance use disorder (SUD) treatment systems from 2013–2022. The manuscript is generally well written, methodologically straightforward, and supported by appropriate data sources. However, it would benefit from stronger framing, greater methodological clarity, more detailed analysis of trends, and a more nuanced discussion connecting the results to existing literature. Several limitations also need more explicit and specific treatment.

Overall, I recommend major revisions to strengthen interpretation, clarity, and methodological transparency.

The aim of the article is not clearly stated in the abstract, and emphasizing it more explicitly would help readers understand the purpose and contribution of the study. Additionally, although the paper claims to analyze trends, it does not clearly describe how the reported values change over time. The revised manuscript should be consistent—either functioning as a high-level summary or offering an account of periodical change.

I also noted that Table 1 does not appear to be referenced in the text. The figures are difficult to read and appear pixelated; higher-resolution images would improve readability.

Regarding lines 309–310, the statement about further research is broad and therefore not very informative. The authors should be more precise about what types of future research are needed. For example, it would be helpful to specify what kinds of studies could translate these descriptive findings into actionable steps to improve interventions and enhance care for this population.

Reviewer #2: Thank you very much for the opportunity to review this interesting article. The subject matter discussed would be, I believe, of interest to the readership of PLOS Mental Health. However, my main concern centres around the analysis of what is a very large dataset, which has the potential to provide significant insight. The study only considers univariate statistics in the analysis, while it would be useful to see, as you say, “national trends of alcohol or substance-induced disorders in the mental health treatment setting alongside substance use disorder cases”. Multivariate analysis would be welcomed here, and I would advise discussing with a statistician the best approach to take to conduct robust analysis. This should lead to a significant revision and strengthening of the discussion section, in which I would advise that you think much more carefully about what the results are and are not showing you, and the potential implications of this.

In summary, while the paper has the potential to offer important insights in this area, more robust analysis and discussion is first required.

**********

what does this mean?). If published, this will include your full peer review and any attached files.

**Do you want your identity to be public for this peer review?**  If you choose “no”, your identity will remain anonymous but your review may still be made public.

For information about this choice, including consent withdrawal, please see our Privacy Policy

Reviewer #1: No

Reviewer #2: No

**********

[NOTE: If reviewer comments were submitted as an attachment file, they will be attached to this email and accessible via the submission site. Please log into your account, locate the manuscript record, and check for the action link "View Attachments". If this link does not appear, there are no attachment files.]

# Author comment: Author response to Decision Letter 1

**DOI:** 10.1371/journal.pmen.0000706.r002

2 Jan 2026

Attachment Submitted filename: Reviewer Response.docx

# Peer review: Decision Letter 1

**DOI:** 10.1371/journal.pmen.0000706.r003

**Authors:** Gellan Karamallah Ramadan Ahmed

22 Jan 2026

PMEN-D-25-00524R1

Alcohol and Substance-Induced Disorder Treatment Cases in Mental Health and Addiction Treatment Facilities from 2013 to 2022: A Descriptive Study

PLOS Mental Health

Dear Dr. Ware,

Thank you for submitting your manuscript to PLOS Mental Health. After careful consideration, we feel that it has merit but does not fully meet PLOS Mental Health’s publication criteria as it currently stands. Therefore, we invite you to submit a revised version of the manuscript that addresses the points raised during the review process.

Please submit your revised manuscript by Feb 21 2026 11:59PM. If you will need more time than this to complete your revisions, please reply to this message or contact the journal office at mentalhealth@plos.org. When you're ready to submit your revision, log on to https://www.editorialmanager.com/pmen/ and select the 'Submissions Needing Revision' folder to locate your manuscript file.

Please include the following items when submitting your revised manuscript:

- A letter that responds to each point raised by the editor and reviewer(s). You should upload this letter as a separate file labeled 'Response to Reviewers'.
- A marked-up copy of your manuscript that highlights changes made to the original version. You should upload this as a separate file labeled 'Revised Manuscript with Track Changes'.
- An unmarked version of your revised paper without tracked changes. You should upload this as a separate file labeled 'Manuscript'.

Guidelines for resubmitting your figure files are available below the reviewer comments at the end of this letter.

We look forward to receiving your revised manuscript.

Kind regards,

Gellan Karamallah Ramadan Ahmed

Academic Editor

PLOS Mental Health

Journal Requirements:

If the reviewer comments include a recommendation to cite specific previously published works, please review and evaluate these publications to determine whether they are relevant and should be cited. There is no requirement to cite these works unless the editor has indicated otherwise.

Please review your reference list to ensure that it is complete and correct. If you have cited papers that have been retracted, please include the rationale for doing so in the manuscript text, or remove these references and replace them with relevant current references. Any changes to the reference list should be mentioned in the rebuttal letter that accompanies your revised manuscript. If you need to cite a retracted article, indicate the article’s retracted status in the References list and also include a citation and full reference for the retraction notice.

Additional Editor Comments (if provided):

[Note: HTML markup is below. Please do not edit.]

Reviewers' comments:

Reviewer's Responses to Questions

**Comments to the Author**

Reviewer #1: All comments have been addressed

Reviewer #2: (No Response)

**********

publication criteria?

Reviewer #1: Yes

Reviewer #2: Partly

**********

3. Has the statistical analysis been performed appropriately and rigorously?

Reviewer #1: Yes

Reviewer #2: No

**********

4. Have the authors made all data underlying the findings in their manuscript fully available (please refer to the Data Availability Statement at the start of the manuscript PDF file)?

The PLOS Data policy

Reviewer #1: Yes

Reviewer #2: Yes

**********

5. Is the manuscript presented in an intelligible fashion and written in standard English?

Reviewer #1: Yes

Reviewer #2: Yes

**********

Reviewer #1: My concerns have been adressed.

Reviewer #2: Thank you for the submission of your revised article. You have clarified that as this is a descriptive study, no further analysis will be done. As per Lesko et al. (2022) https://doi.org/10.1093/aje/kwac115 which has guided your study, further detail is still required as to the action(s) that might be informed by the results that you have found.

Your interpretation also requires greater depth, specifically regarding how the aforementioned results could inform or improve future practice and outcomes and the future research that is required for this to be feasible. For example, why would it be important for future research to examine inpatient compared to outpatient data? What would the clinical significance be to this?

**********

what does this mean?). If published, this will include your full peer review and any attached files.

**Do you want your identity to be public for this peer review?**  If you choose “no”, your identity will remain anonymous but your review may still be made public.

For information about this choice, including consent withdrawal, please see our Privacy Policy

Reviewer #1: No

Reviewer #2: No

**********

[NOTE: If reviewer comments were submitted as an attachment file, they will be attached to this email and accessible via the submission site. Please log into your account, locate the manuscript record, and check for the action link "View Attachments". If this link does not appear, there are no attachment files.]

# Author comment: Author response to Decision Letter 2

**DOI:** 10.1371/journal.pmen.0000706.r004

5 Feb 2026

Attachment Submitted filename: Response to Reviewers.docx

# Peer review: Decision Letter 2

**DOI:** 10.1371/journal.pmen.0000706.r005

**Authors:** Gellan Karamallah Ramadan Ahmed

22 Apr 2026

PMEN-D-25-00524R2

Alcohol and Substance-Induced Disorder Treatment Cases in Mental Health and Addiction Treatment Facilities from 2013 to 2022: A Descriptive Study

PLOS Mental Health

Dear Dr. Ware,

Thank you for submitting your manuscript to PLOS Mental Health. After careful consideration, we feel that it has merit but does not fully meet PLOS Mental Health’s publication criteria as it currently stands. Therefore, we invite you to submit a revised version of the manuscript that addresses the points raised during the review process.

Please submit your revised manuscript by May 22 2026 11:59PM. If you will need more time than this to complete your revisions, please reply to this message or contact the journal office at mentalhealth@plos.org. When you're ready to submit your revision, log on to https://www.editorialmanager.com/pmen/ and select the 'Submissions Needing Revision' folder to locate your manuscript file.

Please include the following items when submitting your revised manuscript:

- A letter that responds to each point raised by the editor and reviewer(s). You should upload this letter as a separate file labeled 'Response to Reviewers'.
- A marked-up copy of your manuscript that highlights changes made to the original version. You should upload this as a separate file labeled 'Revised Manuscript with Track Changes'.
- An unmarked version of your revised paper without tracked changes. You should upload this as a separate file labeled 'Manuscript'.

Guidelines for resubmitting your figure files are available below the reviewer comments at the end of this letter.

As the corresponding author, your ORCID iD is verified in the submission system and will appear in the published article. PLOS supports the use of ORCID, and we encourage all coauthors to register for an ORCID iD and use it as well. Please encourage your coauthors to verify their ORCID iD within the submission system before final acceptance, as unverified ORCID iDs will not appear in the published article. *Only*  the individual author can complete the verification step; PLOS staff *cannot*  verify ORCID iDs on behalf of authors.

We look forward to receiving your revised manuscript.

Kind regards,

Gellan Karamallah Ramadan Ahmed

Academic Editor

PLOS Mental Health

**Journal Requirements:**

If the reviewer comments include a recommendation to cite specific previously published works, please review and evaluate these publications to determine whether they are relevant and should be cited. There is no requirement to cite these works unless the editor has indicated otherwise.

Please review your reference list to ensure that it is complete and correct. If you have cited papers that have been retracted, please include the rationale for doing so in the manuscript text, or remove these references and replace them with relevant current references. Any changes to the reference list should be mentioned in the rebuttal letter that accompanies your revised manuscript. If you need to cite a retracted article, indicate the article’s retracted status in the References list and also include a citation and full reference for the retraction notice.

**Additional Editor Comments (if provided):**

[Note: HTML markup is below. Please do not edit.]

Reviewers' comments:

Reviewer's Responses to Questions

**Comments to the Author**

Reviewer #3: (No Response)

Reviewer #4: (No Response)

**********

publication criteria?

Reviewer #3: Yes

Reviewer #4: Yes

**********

3. Has the statistical analysis been performed appropriately and rigorously?

Reviewer #3: Yes

Reviewer #4: Yes

**********

4. Have the authors made all data underlying the findings in their manuscript fully available (please refer to the Data Availability Statement at the start of the manuscript PDF file)?

The PLOS Data policy

Reviewer #3: Yes

Reviewer #4: Yes

**********

5. Is the manuscript presented in an intelligible fashion and written in standard English?

Reviewer #3: Yes

Reviewer #4: Yes

**********

**Reviewer #3:**  Lines 89 to 93: ‘Due to this gap, one study analyzed the Substance 90 Abuse and Mental Health Services Administration’s Treatment Episode Data Set 91 Admissions [15], reviewing all cases identified as having a “substance-induced disorder” 92 from 1992 to 2022, excluding cases with an “alcohol-induced disorder" unless 93 examining trends of these diagnoses [16]’. The beginning of this argument ‘Due to this gap’ is weak. Kindly rephrase.

Lines 104- 111: ‘[a] Identify the percentage of cases flagged as having 105 alcohol-induced disorders or substance-induced disorders in mental health and 106 substance use disorder treatment settings, [b.] Examine the prevalence of other mental 107 health diagnoses, such as anxiety and depressive disorders, among cases with alcohol108 induced disorders or substance-induced disorders in mental health treatment, and [c.] 109 Examine the prevalence of substances used, such as alcohol and cannabis, among 110 cases with alcohol-induced disorders or substance-induced disorders in mental health 111 treatment’. You do not need to enumerate your objectives. I will advise that you report the main study objective and other specific study objectives can be discussed in the ‘Discussion section’.

Lines 225-228: ‘Building upon the research by Fedotov and Shustov [16], which examined substance226 induced disorder treatment in the addiction setting, this is the first study to analyze 227 alcohol-induced and substance-induced disorders in national treatment records for 228 mental health and substance use issues in the United States’. This is not necessary here. The statement was mentioned earlier in the Introduction section (lines 95- 100). The first paragraph of your discussion section should report the main findings of your study.

Lines 343- 359: ‘Conclusion’. Your conclusion is weak. You do not have to repeat your results in the conclusion. You are only to provide your readers with a summary of the interesting findings or novel findings of your research, not necessarily repeating figures from the results. Recommend in one or two succinct paragraphs and maybe make a key recommendation for future research.

**Reviewer #4:**  Address

Thank you for submitting a revised manuscript.

Summary

This is a descriptive study that assesses alcohol and substance-induced disorder treatment cases in mental health and addiction treatment facilities in the USA from 2013 to 2022. Secondary data was obtained from the Mental Health Client-Level Data and the Treatment Episode Data Set Admissions. Overall, the study is academically and clinically relevant. However, there are a few concerns.

General comments

The term “flagged”, though correct, should be reconsidered. Terms such as “identified”, implicated, etc., are more suitable for a study of this nature.

Also, there are a few grammatical errors that require the author’s attention. Example:

“For example, persons presenting to treatment…” Page 7; line 242

The author should kindly go through the entire manuscript and make the necessary corrections.

Furthermore, some of the sentences are quite long and difficult to read. Example:

“To further explore clinical diagnostic considerations, this study also examines mental health diagnoses reported in those receiving mental health treatment and substances used among individuals with an alcohol or substance-induced disorder who received treatment in either setting between 2013 and 2022.” Page 3; lines 100-103

The long sentences should be made shorter, if possible.

Finally, it will be insightful to know the following trends:

a. Mental disorder with the highest prevalence for each year (between 2013 and 2022).

b. The age group with the highest prevalence of “substance-induced mental disorder” and “alcohol-induced mental disorder” for each year (between 2013 and 2022).

c. Most reported substances for each year (between 2013 and 2022).

d. Sex and race distribution for a, b, and c for each year (between 2013 and 2022).

This will inform policy-makers whether the implicated age group, mental disorders, and substances with the highest prevalence are changing over the years, and advise the design of further studies and interventions.

The author can ignore the final point if it is out of the study’s scope.

Abstract

The abstract provides a sufficient summary of the background, methods, results, and conclusion of the study.

Introduction

“The mental health symptoms resulting from substance-induced disorders are particularly unique, as clinicians must address both the emerging psychological issues and the underlying substance use. In fact, individuals with substance-induced mental health symptoms, such as substance-induced psychosis [5-12], require treatment that considers both mental health and substance use concerns.” Page 3; lines 78-83

The highlighted statement appears to give clarity to the preceding statement. However, the terms and construct make it look similar to the preceding statement. The author can cite specific disorders to better clarify the preceding statement. See below:

“The mental health symptoms resulting from substance-induced disorders are particularly unique, as clinicians are required to address both the emerging psychological issues and the underlying substance use. For example, in the management of alcohol-induced psychotic disorder, it is recommended that the treatment address the psychotic disorder and alcohol use concern.”

Or

“The mental health symptoms resulting from substance-induced disorders are particularly unique, as clinicians are required to address both the emerging psychological issues and the underlying substance use. For example, in the management of cannabis-induced psychotic disorder, it is recommended that the treatment address the psychotic disorder and the concern of cannabis use.”

“Given the unique nature of substance-induced disorders, it is reasonable that facilities specializing in treating mental health disorders, substance use disorders, or both may encounter individuals with these conditions. However, the literature lacks descriptive studies [13, 14] on substance-induced disorder cases within both mental health and substance use treatment settings, alongside clinical characteristics such as substances used and mental health diagnoses.” Page 3; lines 84-89

I humbly submit that the statement above can be improved. I appreciate the intent of the author in trying to link the substance-induced mental disorders with the settings in which they are managed. However, the construction makes it difficult to get the correlation. Also, the succeeding sentence on descriptive studies can be re-examined. The author should kindly consider the construction of the statement above to improve the flow between the sentences and to clearly indicate the import of the statement. Finally, references 13 and 14 talk about the framework for descriptive epidemiology and heart failure epidemiology, respectively. The context of the limited descriptive studies mentioned in those papers, in my humble opinion, is related to their subject of study. While I acknowledge that references 13 and 14 support the author’s argument that there is a (general) lack of descriptive studies, it will be more appropriate to demonstrate, specifically, that there is a lack of descriptive studies on alcohol and substance-induced disorder treatment cases in mental health and addiction treatment facilities. This will further justify the current study.

Materials and Methods

Efforts to access data were unsuccessful. The final decision concerning the validity of the findings, based on the data, rests with the editor.

Results

• Figures

Some of the figures, as acknowledged by the author, are blurry and difficult to read. The author may consider alternative ways to improve the visuals, if possible.

Discussion

The author reports on the prevalence of Mental Health and Substance Use Disorder Treatment Cases, Substances Used, and Mental Health Disorders under “Results”. However, it is difficult to link the author’s interpretation and reasons for the observed findings to the reported results. The author should please dwell on available studies to overtly explain why the observed “prevalences” of the alcohol-induced disorder, substance-induced disorder, implicated mental disorders, and specific substances were noticed in this current study. This can be done in the light of other national, regional, continental, or global studies.

Limitation

“Future studies examining different levels of care are needed, as outpatient treatment is typically provided to persons with less severe conditions or more stable environmental support than those in inpatient or residential treatment. Therefore, these different levels of care may often represent different client populations receiving services.” Page 9; lines 330-334

In my humble opinion, the highlighted statement appears redundant.

“While this study identifies cases flagged as having an alcohol- or substance-induced disorder, the true prevalence of these conditions cannot be determined in either dataset.” Page 9; lines 338-340.

While the sincerity of the author, with respect to the limitations of the study, is acknowledged, the inability to identify the true prevalence of the parameters being assessed raises some concern. This is because the unanswered gap (prevalence) is part of the aims of the study.

Conclusion

It will be helpful to include the findings of the common mental health disorders and implicated substances identified in the study as part of the conclusion. This is because these parameters are part of the aim.

References

“Substance-induced disorders are a category of conditions caused by substance use” (ref). Page 3; line 75

“In fact, individuals with substance-induced mental health symptoms, such as substance-induced psychosis [5-12], require treatment that considers both mental health and substance use concerns” (ref). Page 3; lines 81-83

“Therefore, managing both the impact of the substance and subsequent mental health symptoms can enhance the care that individuals receive (ref). Variability in the conditions that are induced and the severity of these substance-induced disorders are impacted by the specific substance or substances that the person uses” (ref). Page 7; lines 249-252

Providing references for these statements will be helpful.

Efforts to verify references 15 and 17 were unsuccessful.

Response to Review Comments

The response to Reviewer 2’s comment can be reconsidered to improve clarity and flow.

**********

what does this mean?). If published, this will include your full peer review and any attached files.

**Do you want your identity to be public for this peer review?**  If you choose “no”, your identity will remain anonymous but your review may still be made public.

For information about this choice, including consent withdrawal, please see our Privacy Policy

Reviewer #3: No

Reviewer #4: No

**********

[NOTE: If reviewer comments were submitted as an attachment file, they will be attached to this email and accessible via the submission site. Please log into your account, locate the manuscript record, and check for the action link "View Attachments". If this link does not appear, there are no attachment files.]

**Figure Resubmissions:**

# Author comment: Author response to Decision Letter 3

**DOI:** 10.1371/journal.pmen.0000706.r006

30 Apr 2026

Attachment Submitted filename: Response_to_Reviewers_auresp_3.docx

# Peer review: Decision Letter 3

**DOI:** 10.1371/journal.pmen.0000706.r007

**Authors:** Gellan Karamallah Ramadan Ahmed

2 Jun 2026

PMEN-D-25-00524R3

Alcohol and Substance-Induced Disorder Treatment Cases in Mental Health and Addiction Treatment Facilities from 2013 to 2022: A Descriptive Study

PLOS Mental Health

Dear Dr. Ware,

Thank you for submitting your manuscript to PLOS Mental Health. After careful consideration, we feel that it has merit but does not fully meet PLOS Mental Health’s publication criteria as it currently stands. Therefore, we invite you to submit a revised version of the manuscript that addresses the points raised during the review process.

Please submit your revised manuscript by Jul 02 2026 11:59PM. If you will need more time than this to complete your revisions, please reply to this message or contact the journal office at mentalhealth@plos.org. When you're ready to submit your revision, log on to https://www.editorialmanager.com/pmen/ and select the 'Submissions Needing Revision' folder to locate your manuscript file.

Please include the following items when submitting your revised manuscript:

- A letter that responds to each point raised by the editor and reviewer(s). You should upload this letter as a separate file labeled 'Response to Reviewers'.
- A marked-up copy of your manuscript that highlights changes made to the original version. You should upload this as a separate file labeled 'Revised Manuscript with Track Changes'.
- An unmarked version of your revised paper without tracked changes. You should upload this as a separate file labeled 'Manuscript'.

Guidelines for resubmitting your figure files are available below the reviewer comments at the end of this letter.

As the corresponding author, your ORCID iD is verified in the submission system and will appear in the published article. PLOS supports the use of ORCID, and we encourage all coauthors to register for an ORCID iD and use it as well. Please encourage your coauthors to verify their ORCID iD within the submission system before final acceptance, as unverified ORCID iDs will not appear in the published article. *Only*  the individual author can complete the verification step; PLOS staff *cannot*  verify ORCID iDs on behalf of authors.

We look forward to receiving your revised manuscript.

Kind regards,

Gellan Karamallah Ramadan Ahmed

Academic Editor

PLOS Mental Health

**Journal Requirements:**

If the reviewer comments include a recommendation to cite specific previously published works, please review and evaluate these publications to determine whether they are relevant and should be cited. There is no requirement to cite these works unless the editor has indicated otherwise.

Please review your reference list to ensure that it is complete and correct. If you have cited papers that have been retracted, please include the rationale for doing so in the manuscript text, or remove these references and replace them with relevant current references. Any changes to the reference list should be mentioned in the rebuttal letter that accompanies your revised manuscript. If you need to cite a retracted article, indicate the article’s retracted status in the References list and also include a citation and full reference for the retraction notice.

**Additional Editor Comments (if provided):**

[Note: HTML markup is below. Please do not edit.]

Reviewers' comments:

Reviewer's Responses to Questions

**Comments to the Author**

Reviewer #3: All comments have been addressed

Reviewer #4: (No Response)

**********

publication criteria?

Reviewer #3: Yes

Reviewer #4: Yes

**********

3. Has the statistical analysis been performed appropriately and rigorously?

Reviewer #3: Yes

Reviewer #4: Yes

**********

4. Have the authors made all data underlying the findings in their manuscript fully available (please refer to the Data Availability Statement at the start of the manuscript PDF file)?

The PLOS Data policy

Reviewer #3: Yes

Reviewer #4: Yes

**********

5. Is the manuscript presented in an intelligible fashion and written in standard English?

Reviewer #3: Yes

Reviewer #4: Yes

**********

**Reviewer #3:**  The manuscript is fair and I think it is good enough for publication.

**Reviewer #4:**  Address

Thank you for submitting a revised manuscript. The effort is acknowledged.

Summary

This is a descriptive study that assesses alcohol and substance-induced disorder treatment cases in mental health and addiction treatment facilities in the USA from 2013 to 2022. Secondary data was obtained from the Mental Health Client-Level Data and the Treatment Episode Data Set Admissions. Overall, the study is academically and clinically relevant. However, there are a few concerns.

General comments

• Page numbers and line numbers:

The author could have included page numbers and line numbers of corrected sections for easy review.

• Presentation

There are a number of statements and paragraphs that are not clear at a glance. Example:

“Although this study explores alcohol and substance-induced disorders in both mental health and substance use disorder treatments, limitations must be acknowledged.” Page 9; lines 321-322

This statement can read:

Despite employing robust statistical tools in exploring alcohol and substance-induced disorders in both mental health and substance use disorder treatments, limitations must be acknowledged.

Also, the flow and train of thoughts in some aspects of the introduction, results, discussion, limitations, and conclusion can be difficult to grasp at a glance. The author may consider involving colleagues to help with proofreading. Their suggestions may greatly enhance the flow and clarity of the presentation.

• Long sentences

I commend the effort to rewrite the long sentence cited as an example in the review comment.

“This study also examines mental health diagnoses reported in those receiving mental health treatment and substances used among individuals with an alcohol or substance-induced disorder between 2013 and 2022.” Page 3; lines 106-109

However, the reworded statement (above) could have been presented this way:

“This study also examines mental health diagnoses reported in those receiving mental health treatment. Further, it assesses substances used among individuals with an alcohol or substance-induced disorder between 2013 and 2022.”

Also, there are several long sentences, which are difficult to read. Example:

“One study analyzed the Substance Abuse and Mental Health Services Administration’s Treatment Episode Data Set Admissions [15], reviewing all cases identified as having a “substance-induced disorder” from 1992 to 2022, excluding cases with an “alcohol-induced disorder" unless examining trends of these diagnoses [16].” Page 3; lines 95-99

The statement above can read:

“A study analyzing the Substance Abuse and Mental Health Services Administration’s Treatment Episode Data Set Admissions [15], reviewed all cases identified as having a substance-induced disorder from 1992 to 2022. However, it excluded cases with an alcohol-induced disorder unless examining trends of these diagnoses [16]

“Another limitation is that the mental health treatment data includes diagnoses of mental health disorders, while the substance use disorder treatment data considers substance use as a primary, secondary, or tertiary clinical factor, making it difficult to analyze these aspects consistently across both datasets.”

The statement above can read:

“Another limitation is that the mental health treatment data includes diagnoses of mental health disorders, while the substance use disorder treatment data considers substance use as a primary, secondary, or tertiary clinical factor. This makes it difficult to consistently analyze both datasets.”

These are a few examples of the long sentences. The author was expected to go through the entire manuscript and reword the long sentences to improve the presentation.

Introduction

“Substance-induced disorders are a category of conditions caused by substance use (ref).” Page 3; line 75

“psychosis symptoms” Page 3; line 83

The phrase can read “psychotic symptoms”.

“However, the literature lacks descriptive studies on substance-induced disorder cases within both mental health and substance use treatment settings, alongside clinical characteristics such as substances used and mental health diagnoses.” Page 3; lines 88-91

The statement above is not clear. Please, is the highlighted phrase referring to comorbid mental health disorders? If so, the author should kindly make it clear. This can be done by rewording the statement or breaking the statement into two or three clear sentences.

“Given the unique nature of substance-induced disorders, facilities specializing in treating mental health and/or substance use disorders may encounter individuals with these conditions. However, the literature lacks descriptive studies on substance-induced disorder cases within both mental health and substance use treatment settings, alongside clinical characteristics such as substances used and mental health diagnoses. Descriptive studies are important to the research literature as they can highlight the prevalence of specific conditions and who is affected by them.” Page 3; lines 86-93

I appreciate the effort in trying to link this paragraph to the preceding one. However, I humbly think a different opening statement will help. The facilities were designed to handle such cases, so they are expected to receive such cases.

Materials and Methods

Response is well noted. Thank you.

Results

Response is well noted. The figures added to the reviewer response appear better. Thank you.

Discussion

The presentation (flow and clarity) of the discussion can be improved. For example, the second paragraph of the “Discussion” talks about the prevalence of the reported substances, if I am not mistaken. However, it is very difficult to correlate the long and winding opening statements of the paragraph to the import of the paragraph. Page 7; lines 245-258

Also, I acknowledge the position of the author concerning the lack of data. However, like all other novel studies, the discussion can be done in the light of other national, regional, continental, or global studies.

However, the final decision to accept the “Discussion” in this current format rests with the editor.

Limitation

Response is well noted. Thank you.

Conclusion

The conclusion is long. Page 10

It is expected to be succinct. It is supposed to include a very brief background of the study, key findings, and recommendations or implications for future studies. This can be done in a few sentences. The conclusion is not supposed to be a rehash of the results (with figures).

References

Some of the issues concerning references were missed in the response. Examples:

“Substance-induced disorders are a category of conditions caused by substance use” (ref). Page 3; line 75

“Therefore, managing both the impact of the substance and subsequent mental health symptoms can enhance the care that individuals receive (ref). Variability in the conditions that are induced and the severity of these substance-induced disorders are impacted by the specific substance or substances that the person uses” (ref). Page 7; lines 255-258

Please, providing references for these statements will be helpful.

**********

what does this mean?). If published, this will include your full peer review and any attached files.

**Do you want your identity to be public for this peer review?**  If you choose “no”, your identity will remain anonymous but your review may still be made public.

For information about this choice, including consent withdrawal, please see our Privacy Policy

Reviewer #3: No

Reviewer #4: No

**********

[NOTE: If reviewer comments were submitted as an attachment file, they will be attached to this email and accessible via the submission site. Please log into your account, locate the manuscript record, and check for the action link "View Attachments". If this link does not appear, there are no attachment files.]

**Figure Resubmissions:**

# Author comment: Author response to Decision Letter 4

**DOI:** 10.1371/journal.pmen.0000706.r008

10 Jun 2026

Attachment Submitted filename: Response_to_Reviewers_auresp_4.docx

# Peer review: Decision Letter 4

**DOI:** 10.1371/journal.pmen.0000706.r009

**Authors:** Andreas Paris

27 Jul 2026

PMEN-D-25-00524R4

Alcohol and Substance-Induced Disorder Treatment Cases in Mental Health and Addiction Treatment Facilities from 2013 to 2022: A Descriptive Study

PLOS Mental Health

Dear Dr. Ware,

Thank you for submitting your manuscript to PLOS Mental Health. After careful consideration, we feel that it has merit but does not fully meet PLOS Mental Health’s publication criteria as it currently stands. Therefore, we invite you to submit a revised version of the manuscript that addresses the points raised during the review process.

The manuscript has been evaluated one reviewer, and their comments are available below.

The reviewer acknowledges that the manuscript has been substantially strengthened and that the principal concerns regarding methodological transparency, global public-health relevance, and interpretation have been satisfactorily addressed.

A small number of issues remain. In particular, references to educational or BMI “differences” should be revised to reflect that the quantitizing procedure identifies descriptive patterns in the distribution of coded content rather than statistically established between-group differences. Claims regarding the international transferability of the findings should also be moderated to distinguish conceptual relevance to wider global policy debates from empirical transferability beyond the specific Finnish study population. Finally, the conclusion should more clearly reflect the balance identified between structural support and individual autonomy and should avoid overstating the extent to which participants’ views align with established effective policies.

Could you please carefully revise the manuscript to address all comments raised?

Please submit your revised manuscript by Aug 26 2026 11:59PM. If you will need more time than this to complete your revisions, please reply to this message or contact the journal office at mentalhealth@plos.org. When you're ready to submit your revision, log on to https://www.editorialmanager.com/pmen/ and select the 'Submissions Needing Revision' folder to locate your manuscript file.

Please include the following items when submitting your revised manuscript:

- A letter that responds to each point raised by the editor and reviewer(s). You should upload this letter as a separate file labeled 'Response to Reviewers'.
- A marked-up copy of your manuscript that highlights changes made to the original version. You should upload this as a separate file labeled 'Revised Manuscript with Track Changes'.
- An unmarked version of your revised paper without tracked changes. You should upload this as a separate file labeled 'Manuscript'.

Guidelines for resubmitting your figure files are available below the reviewer comments at the end of this letter.

As the corresponding author, your ORCID iD is verified in the submission system and will appear in the published article. PLOS supports the use of ORCID, and we encourage all coauthors to register for an ORCID iD and use it as well. Please encourage your coauthors to verify their ORCID iD within the submission system before final acceptance, as unverified ORCID iDs will not appear in the published article. *Only*  the individual author can complete the verification step; PLOS staff *cannot*  verify ORCID iDs on behalf of authors.

We look forward to receiving your revised manuscript.

Kind regards,

Andreas Paris, PhD

Associate Editor

PLOS Mental Health

**Journal Requirements:**

If the reviewer comments include a recommendation to cite specific previously published works, please review and evaluate these publications to determine whether they are relevant and should be cited. There is no requirement to cite these works unless the editor has indicated otherwise.

Please review your reference list to ensure that it is complete and correct. If you have cited papers that have been retracted, please include the rationale for doing so in the manuscript text, or remove these references and replace them with relevant current references. Any changes to the reference list should be mentioned in the rebuttal letter that accompanies your revised manuscript. If you need to cite a retracted article, indicate the article’s retracted status in the References list and also include a citation and full reference for the retraction notice.

**Additional Editor Comments (if provided):**

[Note: HTML markup is below. Please do not edit.]

Reviewers' comments:

Reviewer's Responses to Questions

**Comments to the Author**

Reviewer #4: (No Response)

**********

publication criteria?

Reviewer #4: Yes

**********

3. Has the statistical analysis been performed appropriately and rigorously?

Reviewer #4: Yes

**********

4. Have the authors made all data underlying the findings in their manuscript fully available (please refer to the Data Availability Statement at the start of the manuscript PDF file)?

The PLOS Data policy

Reviewer #4: Yes

**********

5. Is the manuscript presented in an intelligible fashion and written in standard English?

Reviewer #4: Yes

**********

**Reviewer #4:**  Address

Thank you for submitting a revised manuscript. The effort is appreciated.

General comments

The comments concerning the long sentences, the structure of the conclusion, and unreferenced statements have been fairly addressed. However, there are a few comments

Introduction

“Given the unique nature of substance-induced disorders, facilities specializing in treating mental health and/or substance use disorders may encounter individuals with these conditions. However, the literature lacks descriptive studies on substance-induced disorder cases within both mental health and substance use treatment settings.” Page 3; lines 86-89

Previous Comment by Reviewer

I appreciate the effort in trying to link this paragraph to the preceding one. However, I humbly think a different opening statement will help. The facilities were designed to handle such cases, so they are expected to receive such cases.

Author’s Response

“Not all mental health or addiction facilities are designed to handle cases with comorbidities, even those that may receive such cases, unfortunately. This opening sentence points to this unfortunate clinical reality.”

Reviewer’s Comment

It appears the author’s response to the comment may be missing some words. It is not clear. However, the statement can be accepted since it does not alter the import of the study.

Discussion

The concerns of the discussion have not been addressed. The final decision to accept the “Discussion” in this current format rests with the editor.

**********

what does this mean?). If published, this will include your full peer review and any attached files.

**Do you want your identity to be public for this peer review?**  If you choose “no”, your identity will remain anonymous but your review may still be made public.

For information about this choice, including consent withdrawal, please see our Privacy Policy

Reviewer #4: No

**********

[NOTE: If reviewer comments were submitted as an attachment file, they will be attached to this email and accessible via the submission site. Please log into your account, locate the manuscript record, and check for the action link "View Attachments". If this link does not appear, there are no attachment files.]

**Figure Resubmissions:**

# Author comment: Author response to Decision Letter 5

**DOI:** 10.1371/journal.pmen.0000706.r010

28 Jul 2026

Attachment Submitted filename: Response_to_Reviewers_auresp_5.docx

# Editor report: Decision Letter 5

**DOI:** 10.1371/journal.pmen.0000706.r011

**Authors:** Karli Montague-Cardoso

11 Aug 2026

Alcohol and Substance-Induced Disorder Treatment Cases in Mental Health and Addiction Treatment Facilities from 2013 to 2022: A Descriptive Study

PMEN-D-25-00524R5

Dear Dr. Ware,

We are pleased to inform you that your manuscript 'Alcohol and Substance-Induced Disorder Treatment Cases in Mental Health and Addiction Treatment Facilities from 2013 to 2022: A Descriptive Study' has been provisionally accepted for publication in PLOS Mental Health.

Before your manuscript can be formally accepted you will need to complete some formatting changes, which you will receive in a follow up email. A member of our team will be in touch with a set of requests.

Please note that your manuscript will not be scheduled for publication until you have made the required changes, so a swift response is appreciated.

IMPORTANT: The editorial review process is now complete. PLOS will only permit corrections to spelling, formatting or significant scientific errors from this point onwards. Requests for major changes, or any which affect the scientific understanding of your work, will cause delays to the publication date of your manuscript.

If your institution or institutions have a press office, please notify them about your upcoming paper to help maximize its impact. If they'll be preparing press materials, please inform our press team as soon as possible -- no later than 48 hours after receiving the formal acceptance. Your manuscript will remain under strict press embargo until 2 pm Eastern Time on the date of publication. For more information, please contact mentalhealth@plos.org.

Thank you again for supporting Open Access publishing; we are looking forward to publishing your work in PLOS Mental Health.

Best regards,

Karli Montague-Cardoso

Staff Editor

PLOS Mental Health

***********************************************************

Reviewer Comments (if any, and for reference):
